# Supplementary material for: Oxylipin metabolism is controlled by mitochondrial β-oxidation during bacterial inflammation
Source: Nat Commun. 2022 Jan 10;13:139. doi: 10.1038/s41467-021-27766-8 (PMC8748967; doi:10.1038/s41467-021-27766-8)
Supplement: Supplementary file 1 — Supplementary Information [file 41467_2021_27766_MOESM1_ESM.pdf]

Oxylipin metabolism is controlled by mitochondrial β-oxidation during bacterial inflammation.

Mariya Misheva,

Daniel A White, and Valerie B O'Donnell

Supplementary Figure 1

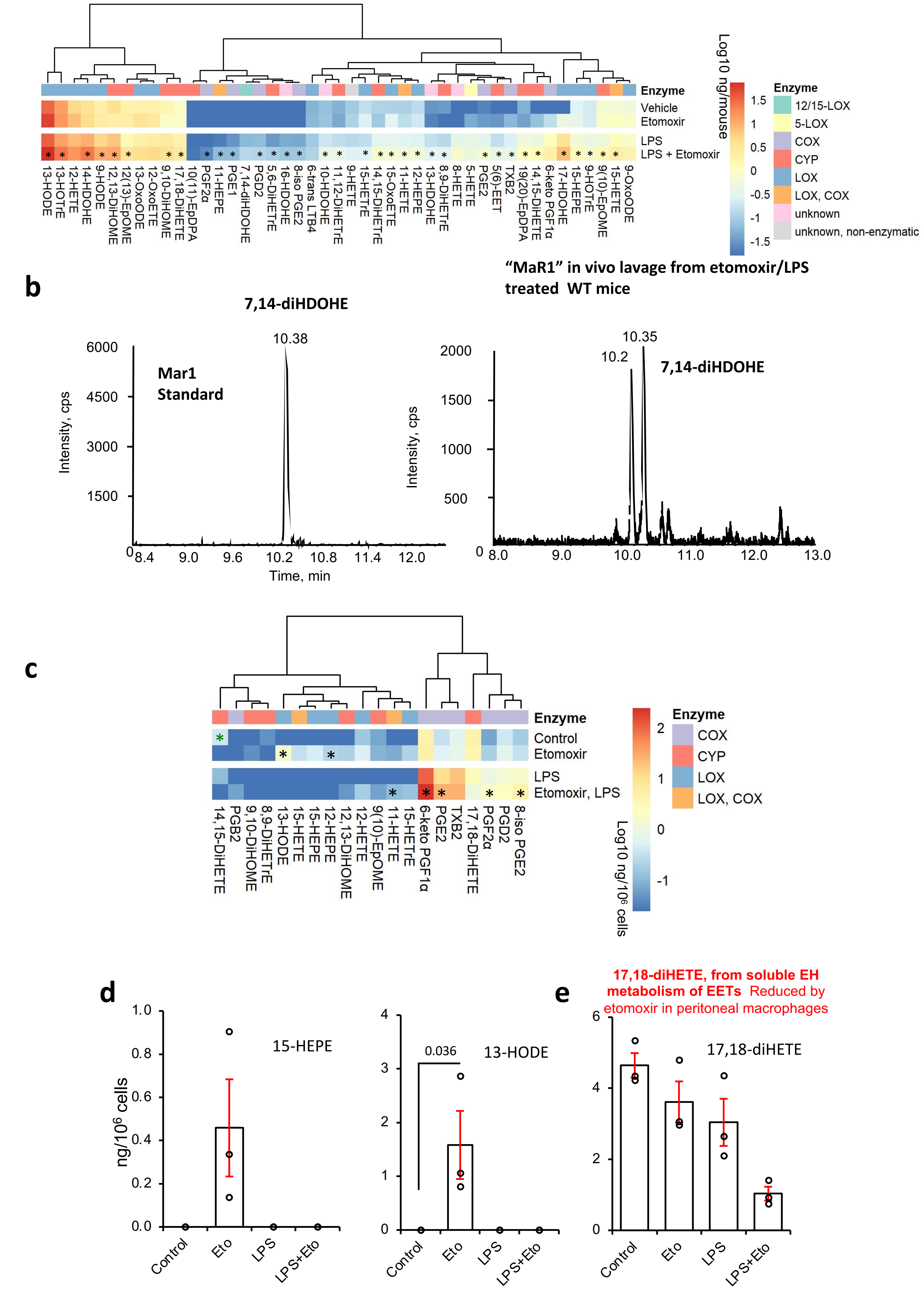

Supplementary Figure 1. Inhibition of CPT1 increases oxylipin levels significantly during inflammation *in vivo,* LC/MS/MS of "Mar1" in mouse peritonitis in response to LPS and the impact of CPT1 inhibition on oxylipins in naïve peritoneal macrophages. *Panel A. Inhibition of CPT1 modulates oxylipin metabolism in vivo.* Wild type mice (female, 7-9 weeks) were injected i.p. with vehicle (PBS), etomoxir (100 μg) or LPS (1 μg). After 6 hrs, lavage was harvested and lipids extracted using SPE then analyzed using LC/MS/MS as outlined in Methods. A heatmap was generated using Pheatmap as described in Methods, with data as log10 ng/total mouse lavage for all oxylipins (n = 10), data is mean +/- SEM, * $p < 0.05$, one way ANOVA with Tukey post hoc test, stats are shown for effect of etomoxir only, where significant. For full ANOVA analysis see Supplementary Data.xls. Tree shows hierarchical clustering. *Panel B. LC/MS/MS of lipid extract from peritoneal lavage (mouse treated with etomoxir and LPS)***.** A lipid with same retention time as Mar1 was detected in lavage from mice treated with etomoxir and LPS *in vivo*, labelled herein as 7,14-diHDOHE. Standard is shown in left panel, mouse lipid in right panel*. Panel C. Heatmap shows that many lipids are elevated when CPT1 is inhibited in vitro*. Peritoneal macrophages were isolated as described in Methods, then cultured in serum-free medium in the presence of etomoxir (25 μM) with/without LPS (100 ng/ml). After 24 hrs, supernatant was harvested and lipids extracted using SPE, then analyzed using LC/MS/MS. A heatmap was generated using Pheatmap as described in Methods, with data as log10 ng/10^6 for all oxylipins (n = 3, data is mean +/- SEM, * $p < 0.01$, one way ANOVA with Tukey post hoc test, stats are shown for effect of etomoxir only, where significant, black: significant elevation with etomoxir, compared to vehicle or LPS alone, green: significant reduction with etomoxir, compared to vehicle or LPS alone). Tree shows hierarchical clustering. *Panels D,E. The impact of LPS and etomoxir on generation of selected oxylipins, measured by LC/MS/MS, in wild type naïve peritoneal macrophages*. Supernatant from peritoneal macrophages was analyzed for oxylipins following culture of macrophages as outlined in methods, for 24 hrs with LPS (100 ng/ml) +/- etomoxir (25 μM). (n = 3, data is mean +/- SEM, separate wells of cells). * $p < 0.05$, one way ANOVA with Tukey post hoc test, stats are shown for effect of etomoxir only, where significant. Where no stars are shown, no significant difference was seen.

Supplementary Figure 2

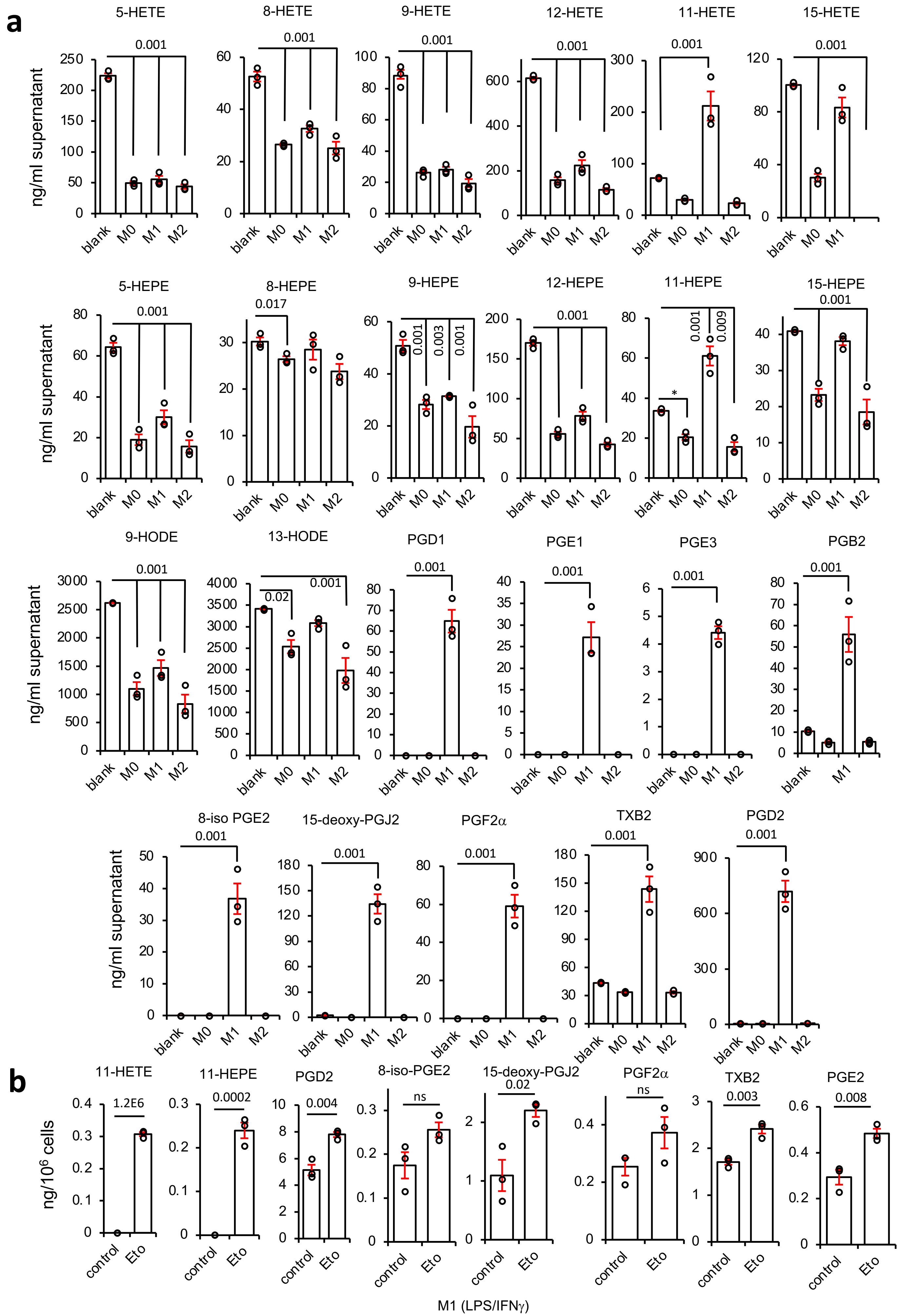

Supplementary Figure 2. BMDM-derived M0, M1 and M2 cells consume diverse oxylipins from serum, while M1 cells consume prostaglandins via CPT1. *Panel A. Serum derived mono-hydroxy oxylipins are metabolized by M0, M1 and M2 cells, while M1 cells generate prostaglandins that are low abundance in serum.* BMDM-derived M0 (M-CSF), M1 (LPS/IFNg), and M2 (IL-4) cells were derived during a 24 hr culture in medium containing 10 % FCS, as described in Methods, then supernatant extracted and analyzed for oxylipins using LC/MS/MS as described in Methods. (n = 3, separate wells of cells). Data is mean +/- SEM, one way ANOVA with Tukey post hoc test, stats are shown for comparison with blank, where significant. Where no stars are shown, no significant difference was seen. *Panel B. M1 cell secretion of prostaglandins is increased by CPT1 inhibition*. BMDM-derived M1 cells were treated in the absence (control) or presence (etomoxir) of etomoxir (25 µM) for 24hr. Here, FCS-free medium was used to avoid contaminating oxylipins from serum (n = 3, mean +/- SEM. Separate wells of cells, with/without etomoxir, Student's T test, two-tailed).

Supplementary Figure 3

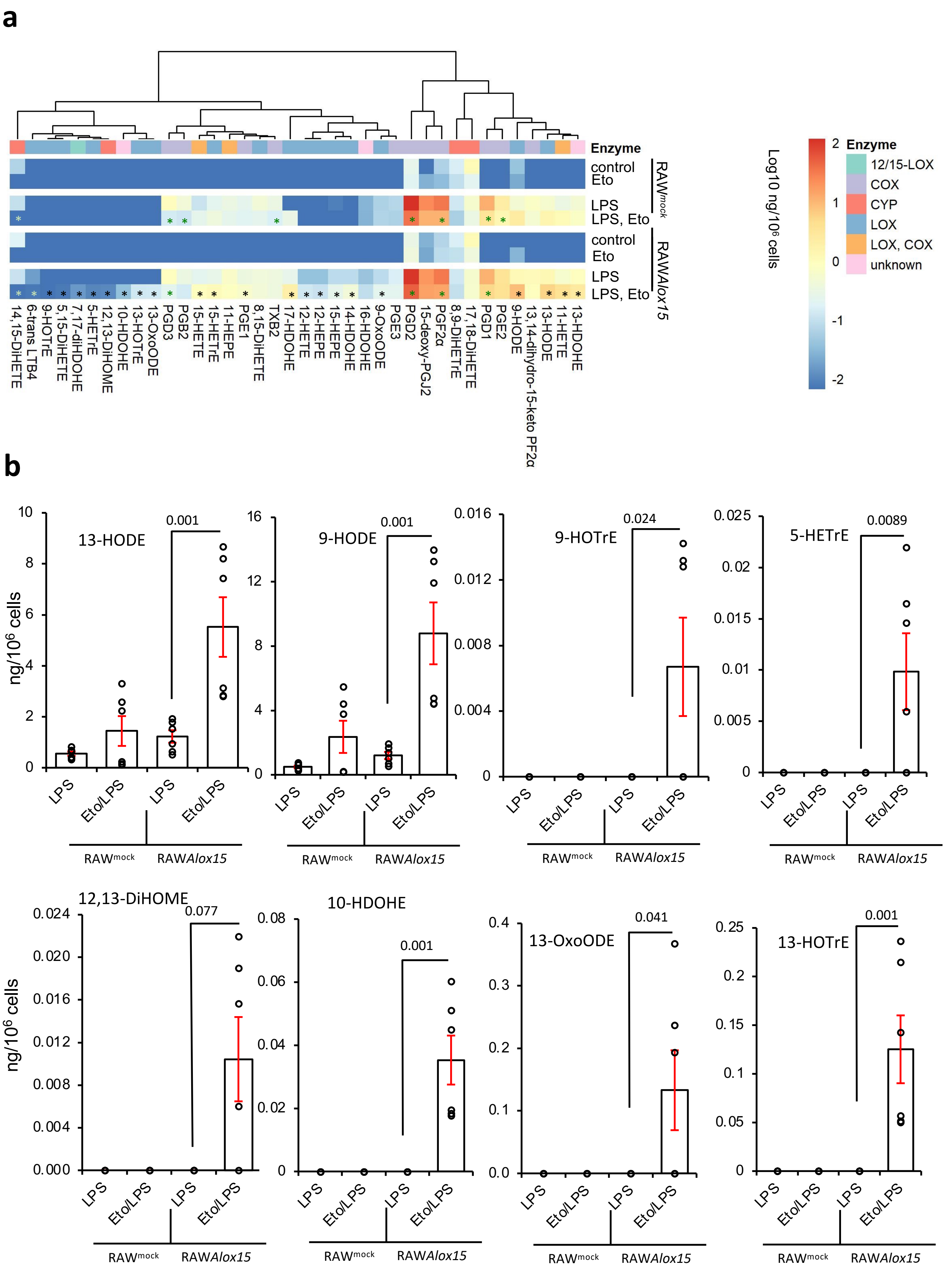

Supplementary Figure 3. Inhibition of CPT1 increases secretion of 12/15-LOX-derived eicosanoids from RAW cells overexpressing *Alox15.* *Panel A.* A heatmap was generated using Pheatmap as described in Methods. Raw^{mock} and Raw*Alox12* cells were cultured with etomoxir (25 mM) and/or LPS (100 ng/ml) for 24 hr before harvest and SPE extraction of supernatant for oxylipin analysis using LC/MS/MS. Data are shown as log10 ng/10^6 values for all oxylipins. black: significant elevation compared to vehicle or LPS alone, green: significant reduction compared to vehicle or LPS alone, $p<0.05$, one way ANOVA with Tukey post hoc test was carried out on the LPS treated samples as a group, stats are shown for effect of etomoxir only, where significant). Tree shows hierarchical clustering. *Panel B. Selected lipids shown in more detail.* (n = 6, separate wells of cells). Data is mean +/- SEM, one way ANOVA with Tukey post hoc test was carried out on the LPS treated samples as a group, stats are shown for effect of etomoxir only, where significant. Where no stars are shown, no significant difference was seen

Supplementary Figure 4

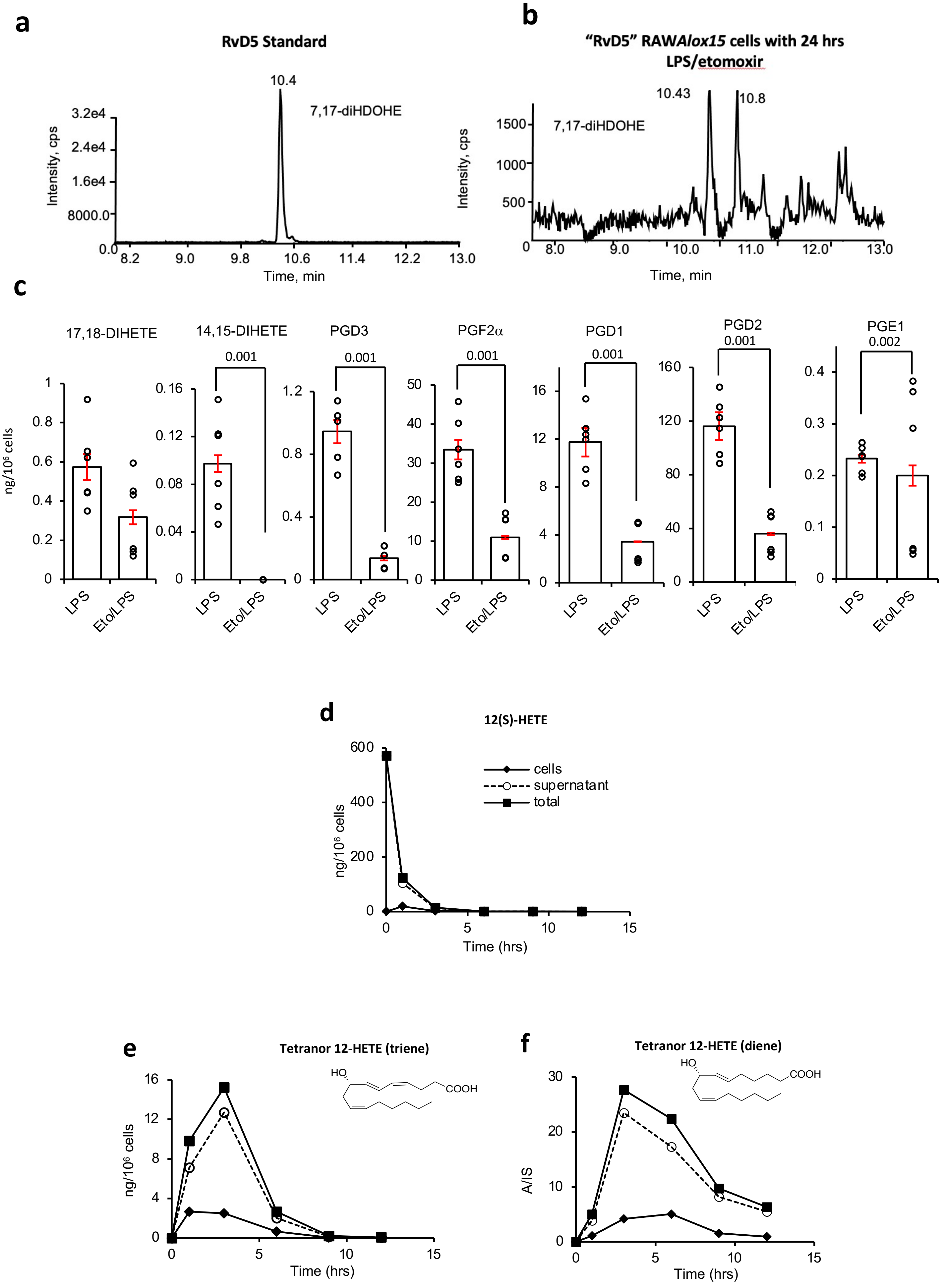

Supplementary Figure 4. Representative chromatograms for 7,17-diHDOHE (“RvD5”) in RAWAlox15 cells, impact of etomoxir on 17,18-di-HETE and PGs in RAW cells, and timecourse of 12-HETE metabolism in RAW cells. *Panel A,B. Supernatant from RAWAlox15 cells treated with LPS/etomoxir contains isomers of 7,17-di-HDOHE*. Cells were cultured with LPS/etomoxir for 24 hrs as in Methods, then supernatant was analyzed for the presence of RvD5 using LC/MS/MS/MS (Panel B). Authentic standard is shown for comparison (Panel A). *Panel C. Etomoxir reduces generation of 17,18-diHETE and PGs from RAW cells*. RAW cells were cultured for 24 hr with/without etomoxir (dose) and LPS (dose) before supernatant was harvested and analyzed using LC/MS/MS as in Methods. (n = 6, mean +/- SEM, separate wells of cells), one way ANOVA with Tukey post hoc test was carried out on the full dataset of RAW^{mock} and RAW*Alox15* but data on RAW^{mock} cells is shown since these lipids are generated by COX or CYP (see Supplementary data). Stats are shown for effect of etomoxir only, where significant. Where no stars are shown, no significant difference was seen. *Panels D-F. 12-HETE is rapidly consumed and converted to two tetranor 12-HETE metabolites by RAW cells*. RAW cells were cultured for up to 12 hr in serum free medium with 1.4 mg 12(S)-HETE added per 10^6 cells. At varying timepoints, samples were harvested and lipids extracted and analyzed using LC/MS/MS as described in Methods.

Supplementary Figure 5

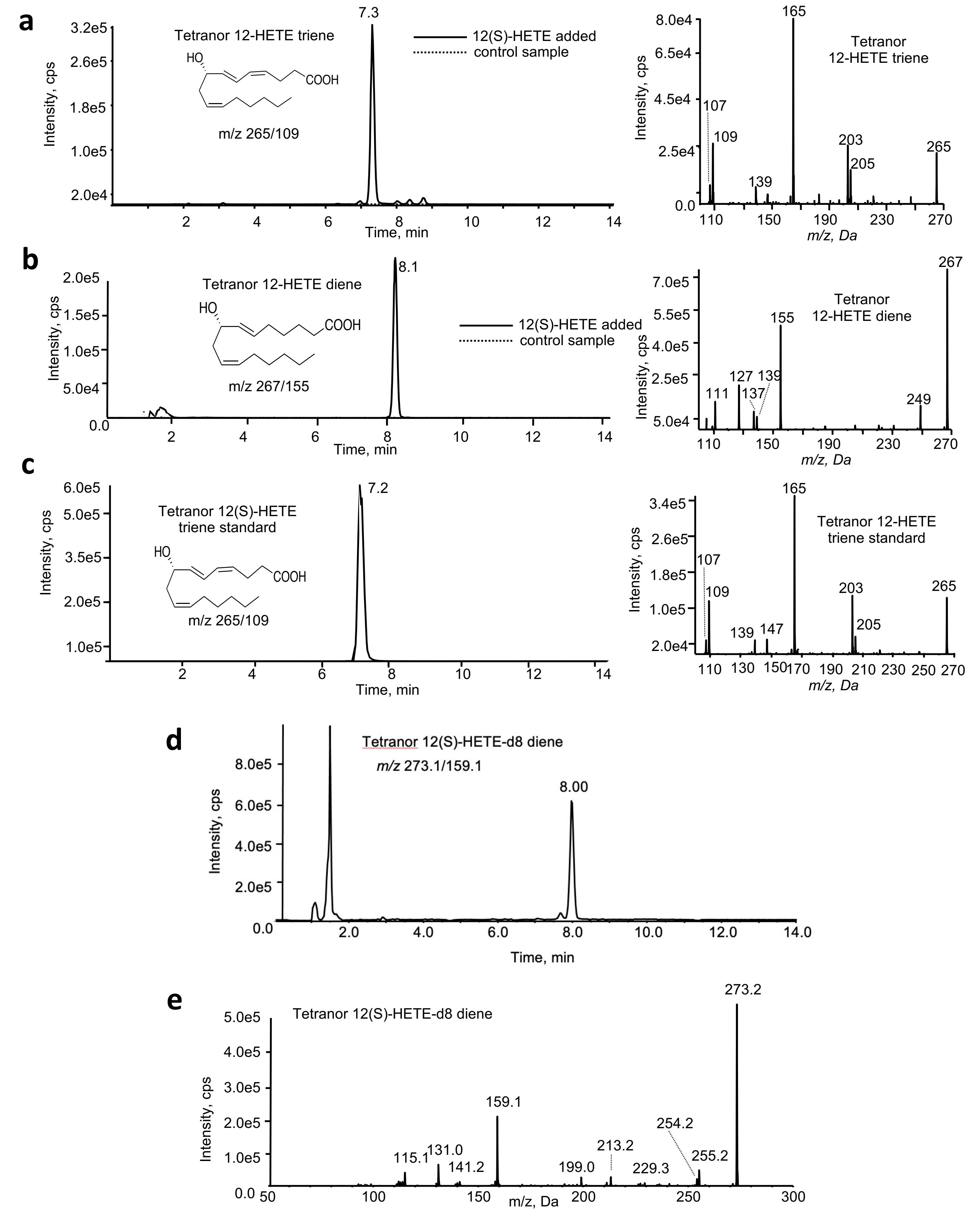

Supplementary Figure 5. Mass spectrometry confirmation of formation of two tetranor 12-HETE metabolites in RAW cells. 12(S)-HETE or 12-HETE-d8 (1.5 mg) were incubated with RAW cells for 3 hrs and then supernatants harvested for LC/MS/MS. *Panels A,B. Formation of tetranor diene and triene 12-HETEs.* A representative sample is shown for each, including chromatogram and MS/MS spectrum, with control (dotted line) and 12(S)-HETE-supplemented (solid line) samples overlaid. *Panel C. MS/MS of the tetranor triene 12-HETE standard*. *Panels D,E. Formation of the 12(S)-HETE-d8 tetranor diene, following supplementation of 12(S)-HETE-d8 to RAW cells (1.5 mg/10^6 cells for 3 hrs),* showing a representative chromatogram (D) and MS/MS spectrum (E).

Supplementary Figure 6

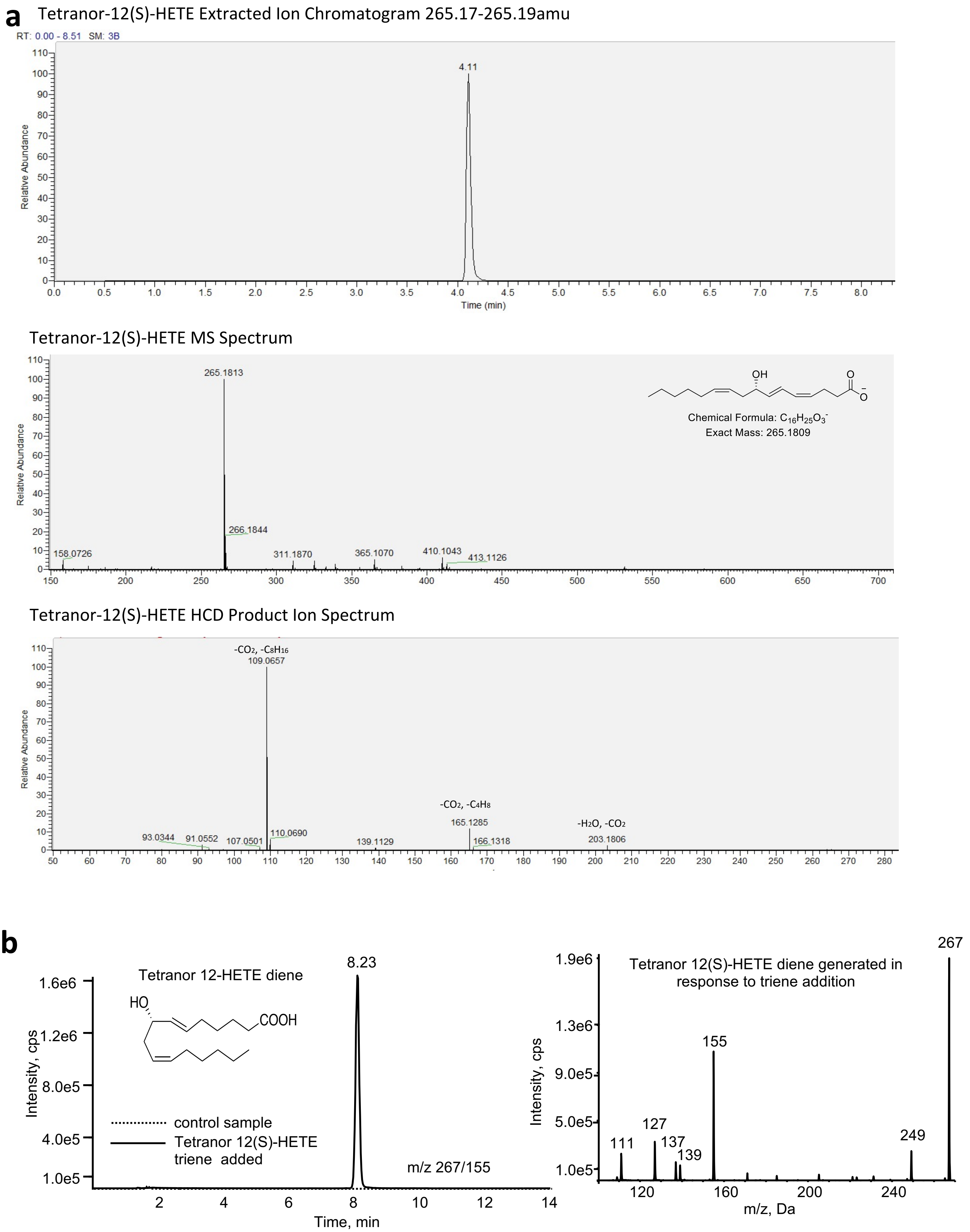

Supplementary Figure 6. High resolution MS/MS of the 12-HETE tetranor triene standard and tetranor triene 12(S)-HETE is converted into the diene isomer in macrophages. *Panel A.* Extracted ion chromatogram, high resolution MS spectrum, and MS/MS spectrum of the tetranor-12(S)-HETE triene standard**.** *Panel B. Addition of tetranor triene 12(S)-HETE leads to formation and secretion of the diene isomer*. 1.2 mg triene standard was incubated per 10^6 RAW cells for 3 hr, then supernatants harvested and analyzed using LC/MS/MS.

Supplementary Figure 7

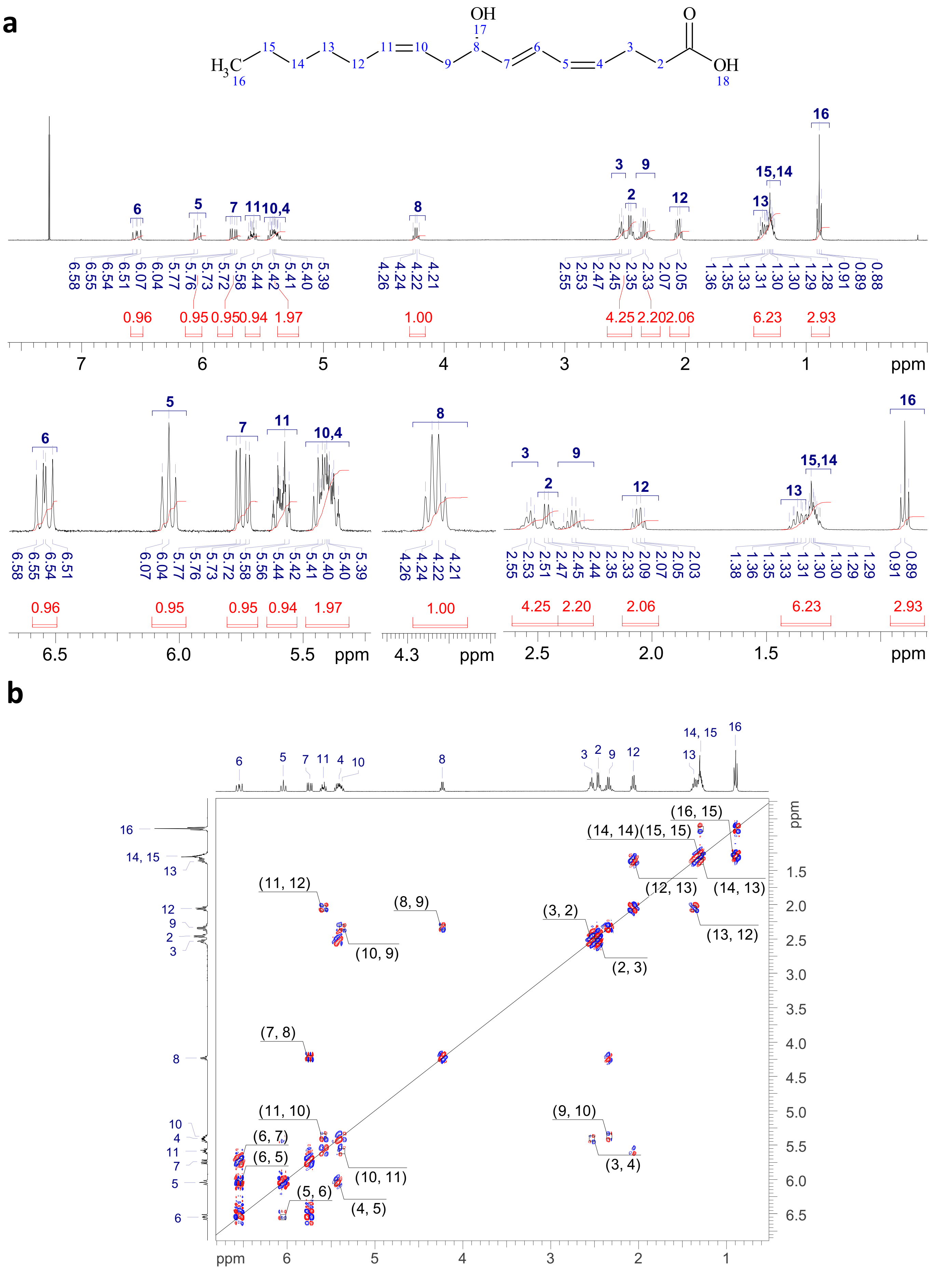

Supplementary Figure 7. ^1H and ^1H-^1H COSY NMR spectra of the tetranor-12(S)-HETE standard. *^1H (Panel A) and ^1H-^1H COSY (Panel B) NMR* demonstrates that the H11 vinyl proton at 5.60 ppm is connected to the methylene H12 protons at 2.05 ppm, which itself is connected back to the alkyl protons at 1.33 ppm. This identifies the 5.60 ppm signal as H11, which correlates with one of the protons that overlap at 5.4 ppm (area of two protons). This 5.4 ppm signal must then be the H10 proton as well as the H4 vinyl proton (which correlates with the H5 vinyl proton and H3 methylene protons).

Supplementary Figure 8

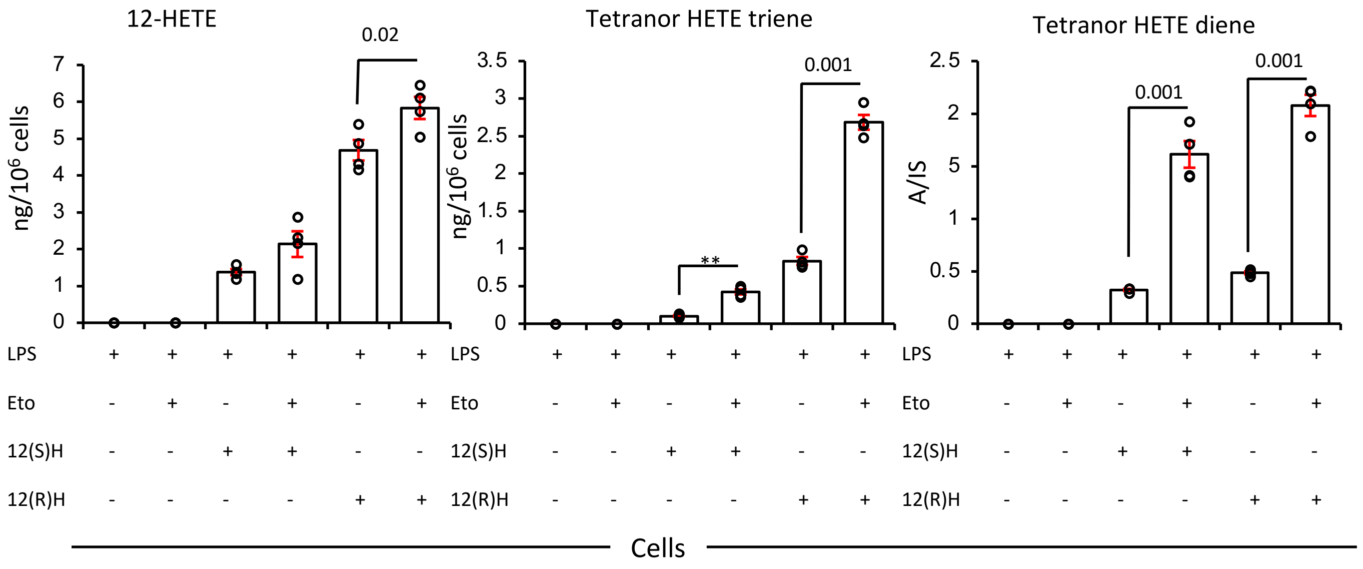

Supplementary Figure 8. CPT1 inhibition prevents metabolism of 12(S) or 12(R)HETE and their tetranor triene and diene metabolites. RAW cells were supplemented with 1.5 mg 12(S) or 12(R)-HETE/10^6 cells for 3 hrs with/without etomoxir (25 mM), then cell pellets were analyzed for levels of 12-HETE and its triene and diene tetranor products using LC/MS/MS (n = 4, mean +/- SEM, separate wells of cells). For all panels, comparisons are with/without etomoxir, one way ANOVA with Tukey post hoc test, stats are shown for effect of etomoxir only, where significant.

Supplementary Figure 9

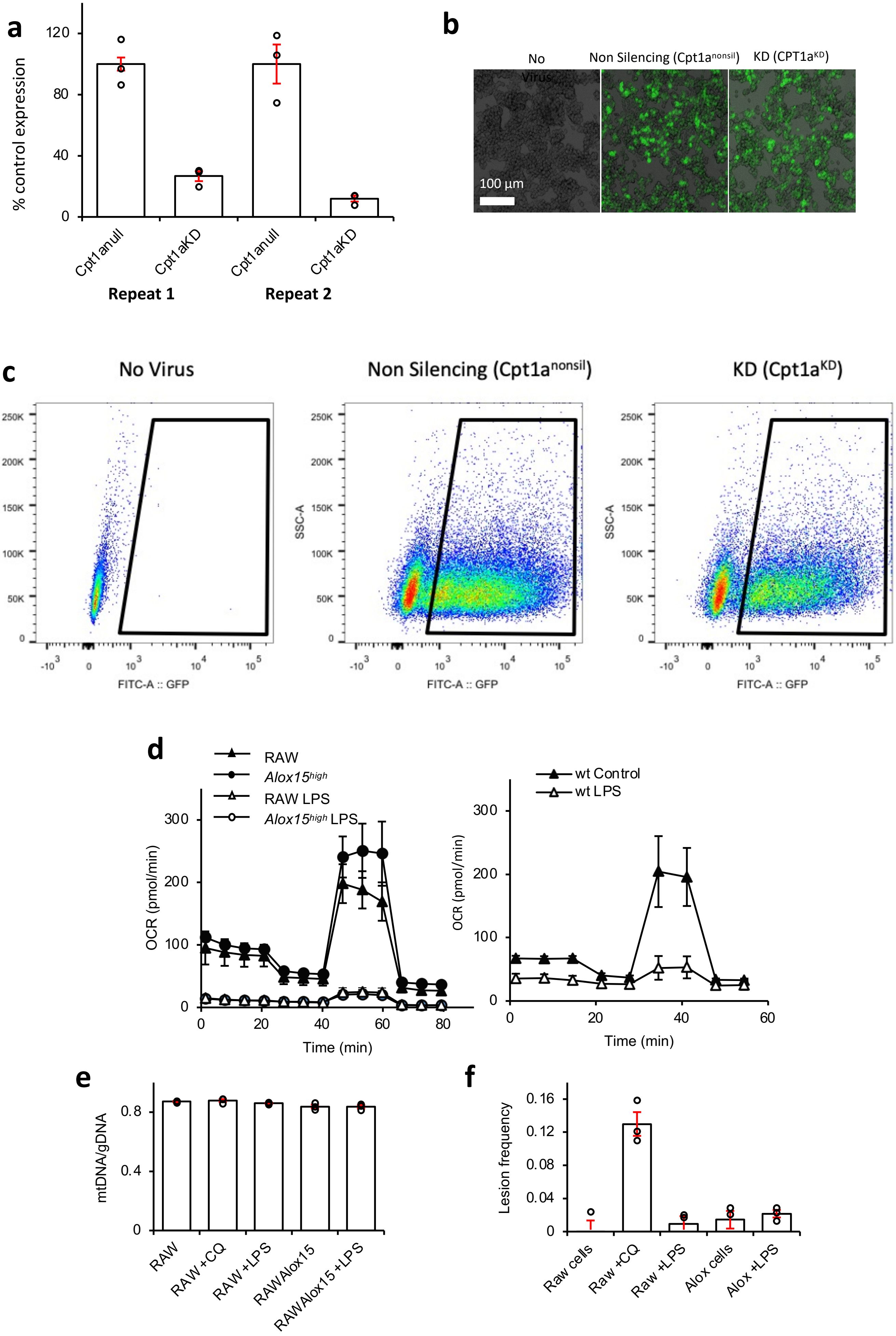

Supplementary Figure 9. Confirmation of *Cpt1a* knockdown in RAW cells and OxPhos is suppressed in LPS-stimulated macrophages *Panel A. Cpt1a expression is suppressed in Cpt1a^{KD} cells versus Cpt1a^{null}.* Bar graph showing the effect of Cpt1a knockdown shRNA on Cpt1a gene expression in RAW264 macrophages, measured by real-time PCR. (n = 3 per group, separate wells of cells, mean +/- SEM), and two repeats are shown. *Panel B. Images of RAW264 cells infected with viruses* (purified by 2.5 μg/ml puromycin) taken on a 10x objective lens using an EVOS microscope (Life Technologies). Image shows transmitted brightfield overlaid with Green fluorescent GFP. Imaging was performed on one occasion as confirmation of infection. *Panel C. Representative images showing GFP expression and sort gates for RAW264 cells infected with viruses using the BD FACS Aria II flow cytometer*. *Panel D. LPS treatment of RAW or naïve peritoneal macrophages reduces mitochondrial OxPhos*. RAW, RAW*Alox15*, or naïve peritoneal macrophages from wild type or *Alox15*$^{-/-}$ mice were treated with LPS (100 ng/ml) for 24 hrs. Mitochondrial function was assessed as described in Methods for peritoneal macrophages (wild type mice) or cell lines (RAW, or *Alox15*^{high}) (n = 5, 4 or 5 respectively, mean +/- SEM). *Panels E,F. LPS doesn't alter mitochondrial DNA or lesion frequency in RAW macrophages.* RAW and RAW*Alox15* cells were mock treated or treated with CQ (40 mM) or LPS (100 ng/ml) for 24 hr. Subsequently their DNA was isolated and used to assess the mtDNA to gDNA ratio (E) and the frequency of lesions in mtDNA (F) using qPCR, one-way ANOVA with Tukey correction, no data were significantly different from RAW samples, (except RAW+CQ, as expected for lesion frequency only, acting as a positive control). Three independent experiments were conducted and a single value for each sample generated in each experiment. These were then averaged (mean +/- SD).

Supplementary Figure 10

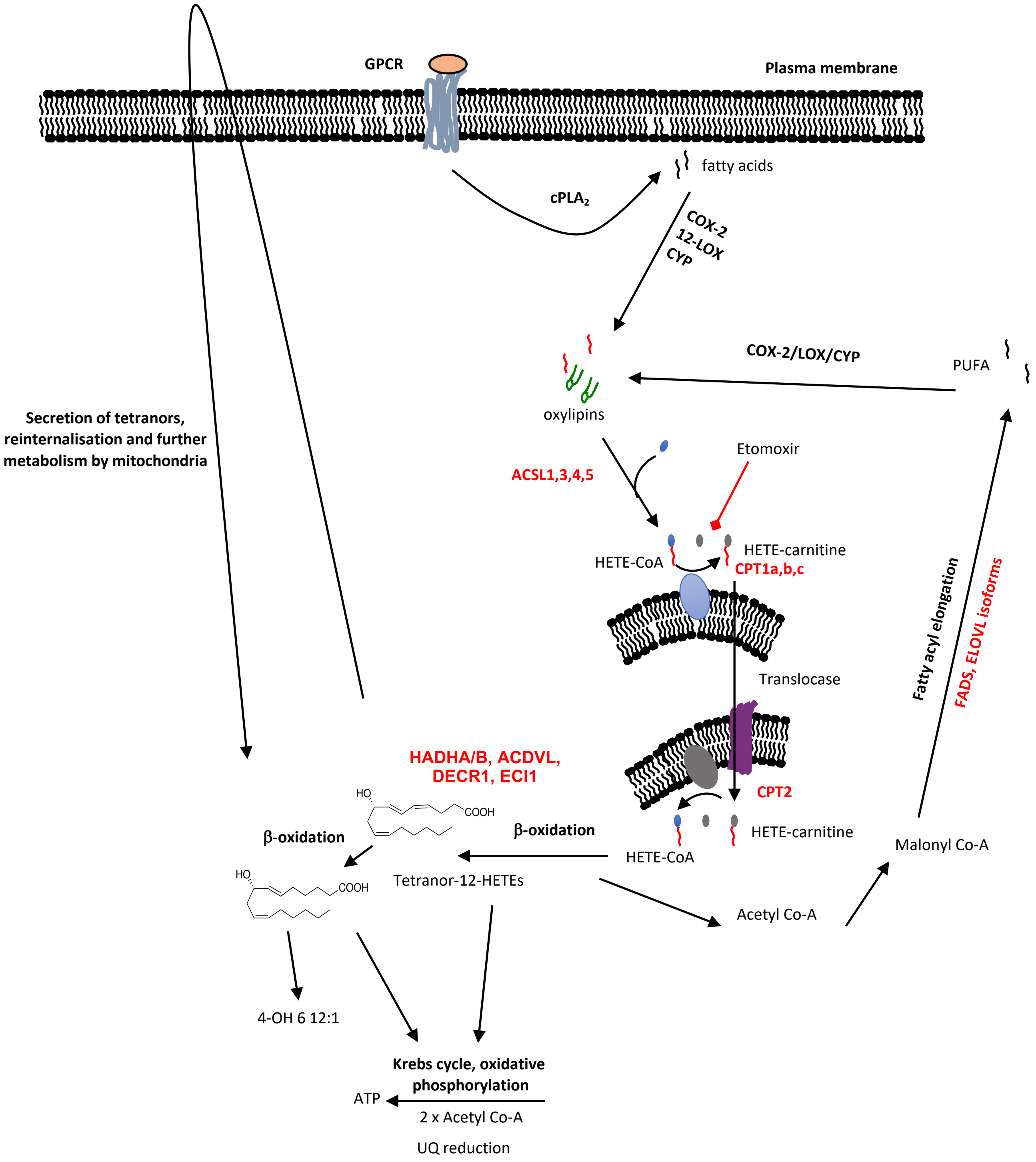

Supplementary Figure 10. Graphic showing formation and mitochondrial metabolism of oxylipins. *Schematic for pathway for mitochondrial metabolism of oxylipins.*

Supplementary Figure 11

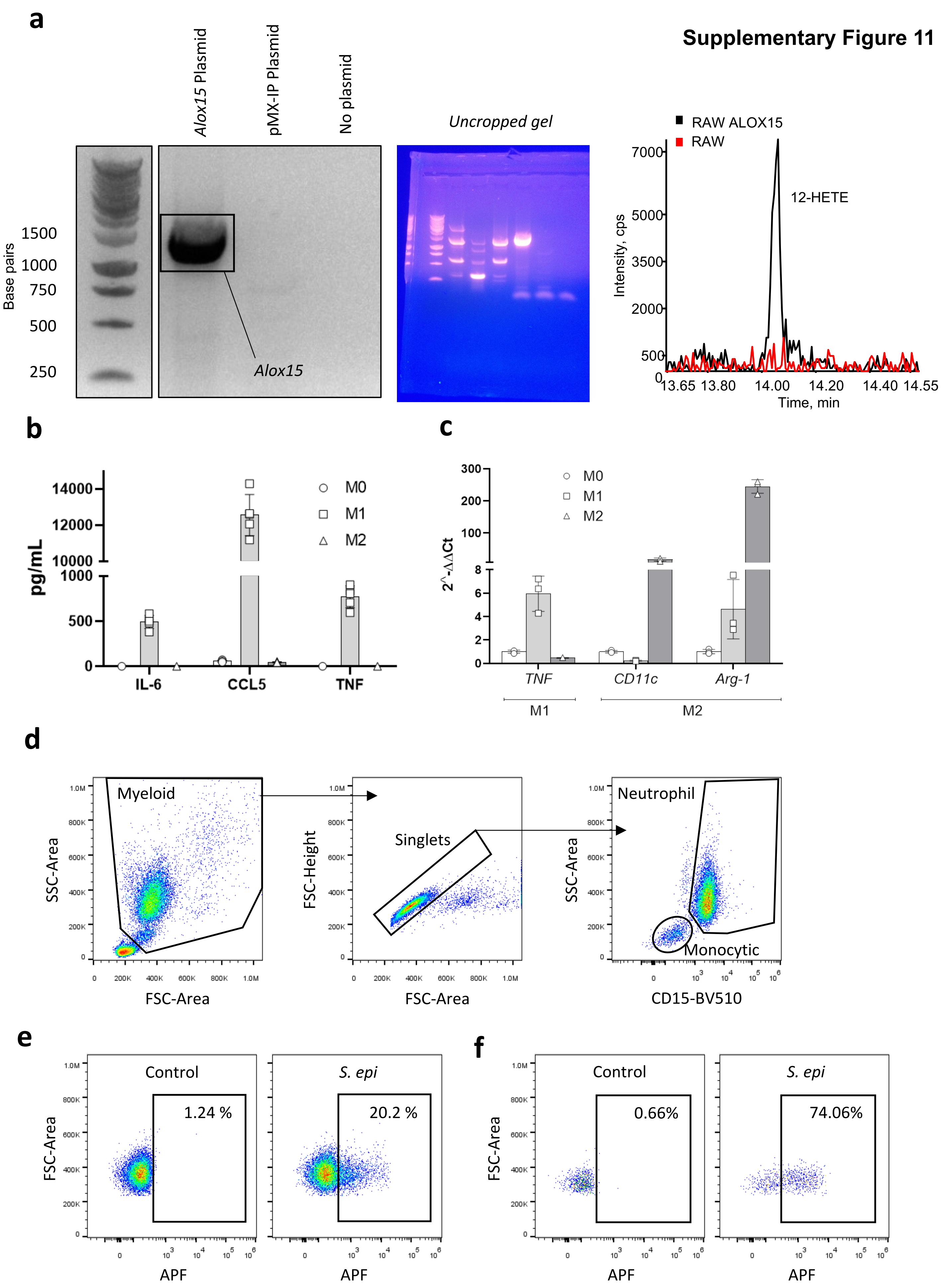

Supplementary Figure 11. Expression of Alox15 in overexpressing RAW cells, confirmation of phenotype in bone marrow derived macrophages, and gating strategy for neutrophils and monocytes in vitro. *Panel A. Expression of Alox15 in RAW cells.* RNA was isolated from the RAW*Alox15*$^{-/-}$ and pMX-IP control cell lines, then converted to cDNA and PCR was performed using primers to amplify *Alox15*. Amplified PCR products were run on a 10% agarose gel and showed that *Alox15* was amplified from the *Alox15* plasmid, but not from the pMX-IP cDNA (plasmid) control, or the no DNA (no plasmid) control, thereby confirming expression as expected. Chromatogram shows generation of 12-HETE in RAWAlox15 cells only, but not mock transfected controls. The full gel is shown (right panel). Unlabelled markers in the left panel (lack of space) are at 10K, 8K, 6K, 5K, 5K, 3K, 2.5K, 2K base pairs. The gel was run once to confirm expression prior to confirmation using the enzyme activity assay. *Panel B. M1 macrophages secrete typical cytokines*. Cytokine production of typical M1 phenotype markers (IL-65, CCL5 and TNF) were determined by ELISA analyses of supernatants post differentiation as described in Methods (n = 5, mean +/- SD). *Panel C. Confirmation of classical and alternative activation in BMDM*. RNA was isolated from activated BMDM, converted to cDNA and then analyzed by real time PCR as described in Methods section. Probes for typical phenotype markers are indicated inset, n = 3, mean +/- SD. *Panels D-F. APF flow cytometric gating strategy.* Representative flow cytometry plots showing the gating strategy for neutrophils and monocytic cells, with a distinctive forward- vs side-scatter profile (FSC vs SSC) that distinguishes them from lymphocytes (Panel D). Neutrophils were further distinguished from eosinophils and monocytes via high expression of CD15. Representative gating for APF^{+ve} neutrophils (Panel E) and monocytes (Panel F) is depicted in the presence or absence of *Staphylococcus epidermidis* (*S. epi*).

Supplementary Figure 12. Representative chromatograms from oxylipin analysis showing typical peaks detected.

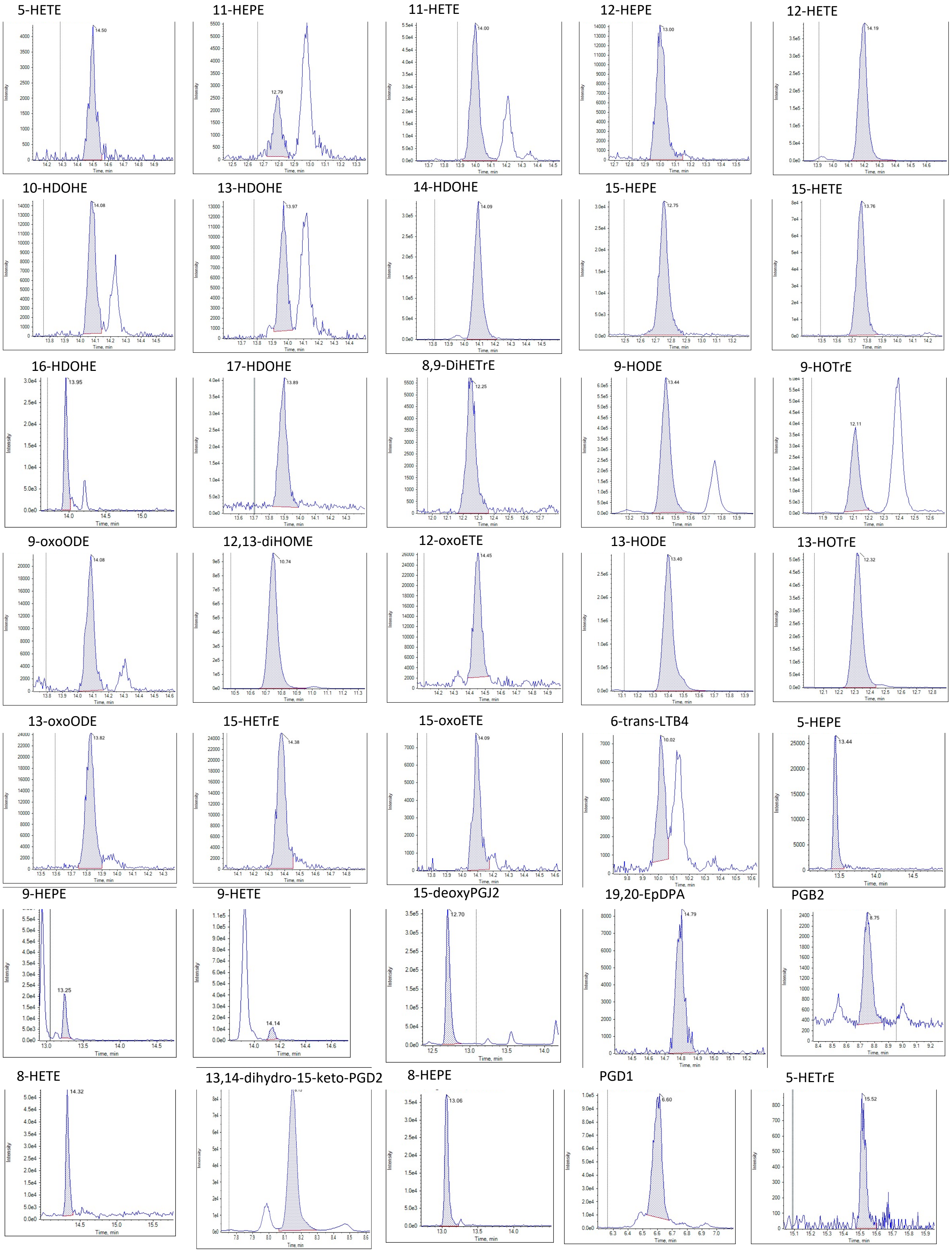

Supplementary Figure 12. **Representative chromatograms from oxylipin analysis showing typical peaks detected.**

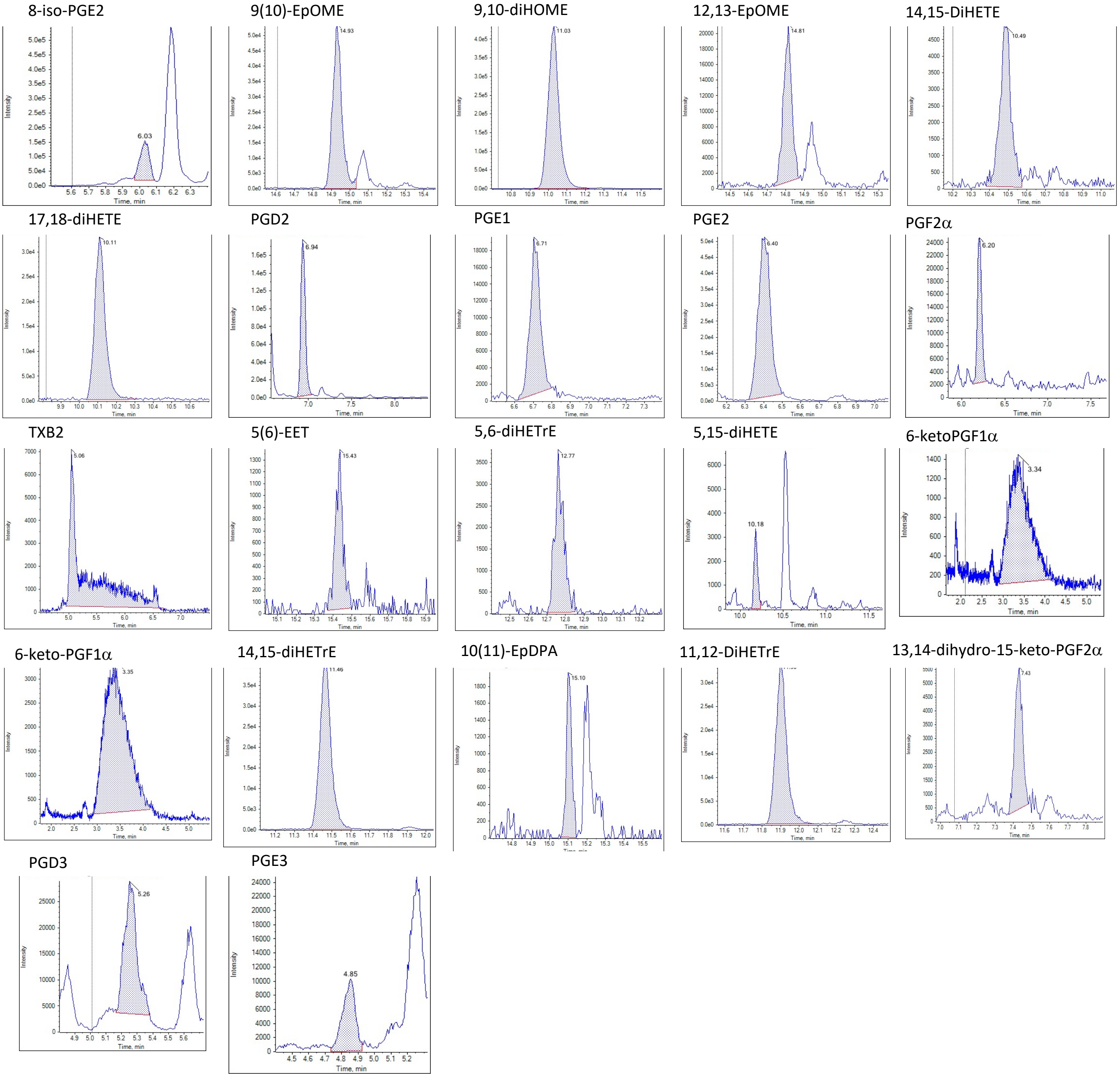

Supplementary Figure 12

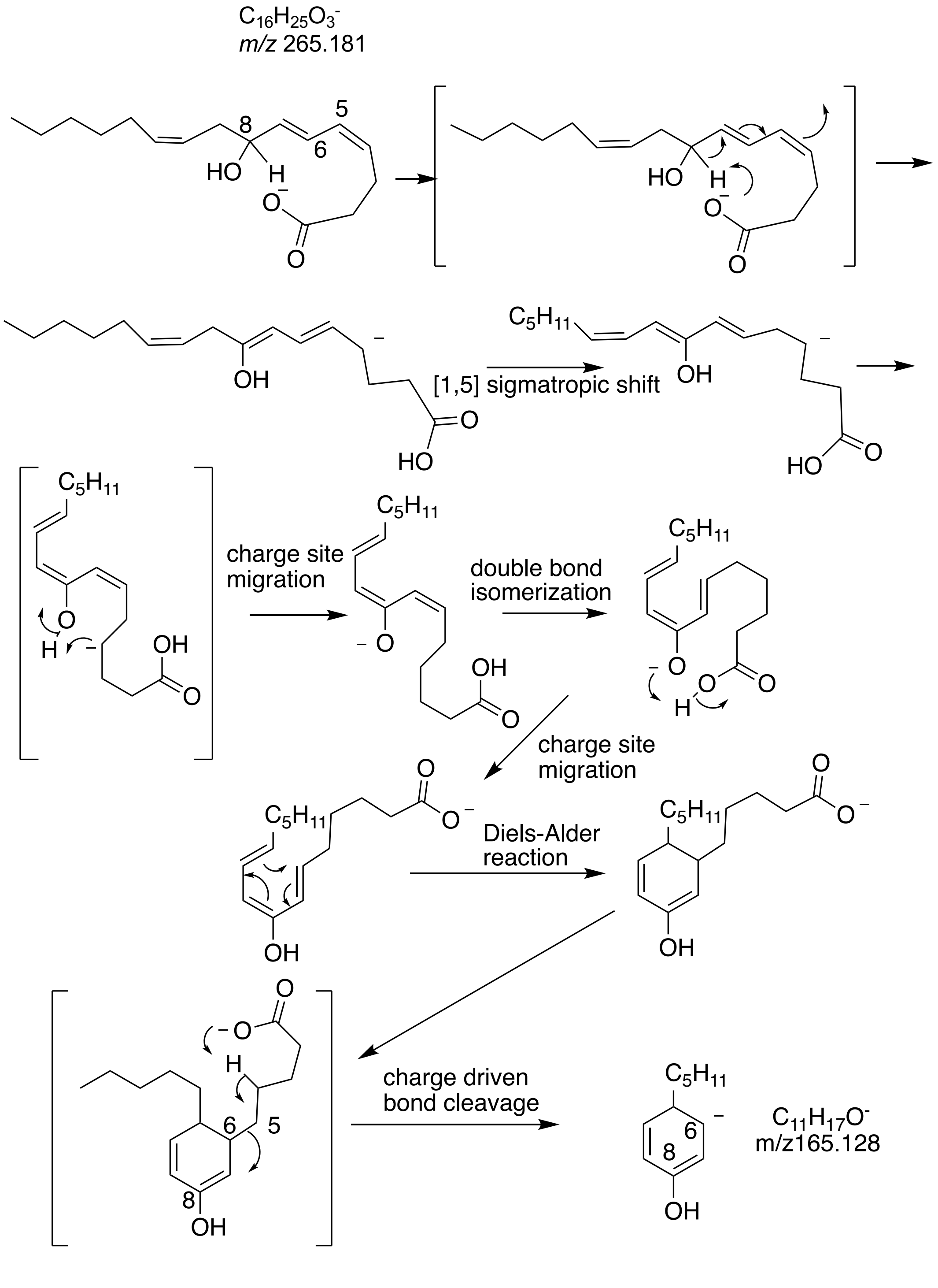

Supplementary Scheme 1. Proposed MS/MS for 8S-hydroxy-4Z,6E,10Z-hexadecatrienoic acid (tetranor triene).

Supplementary Figure 13

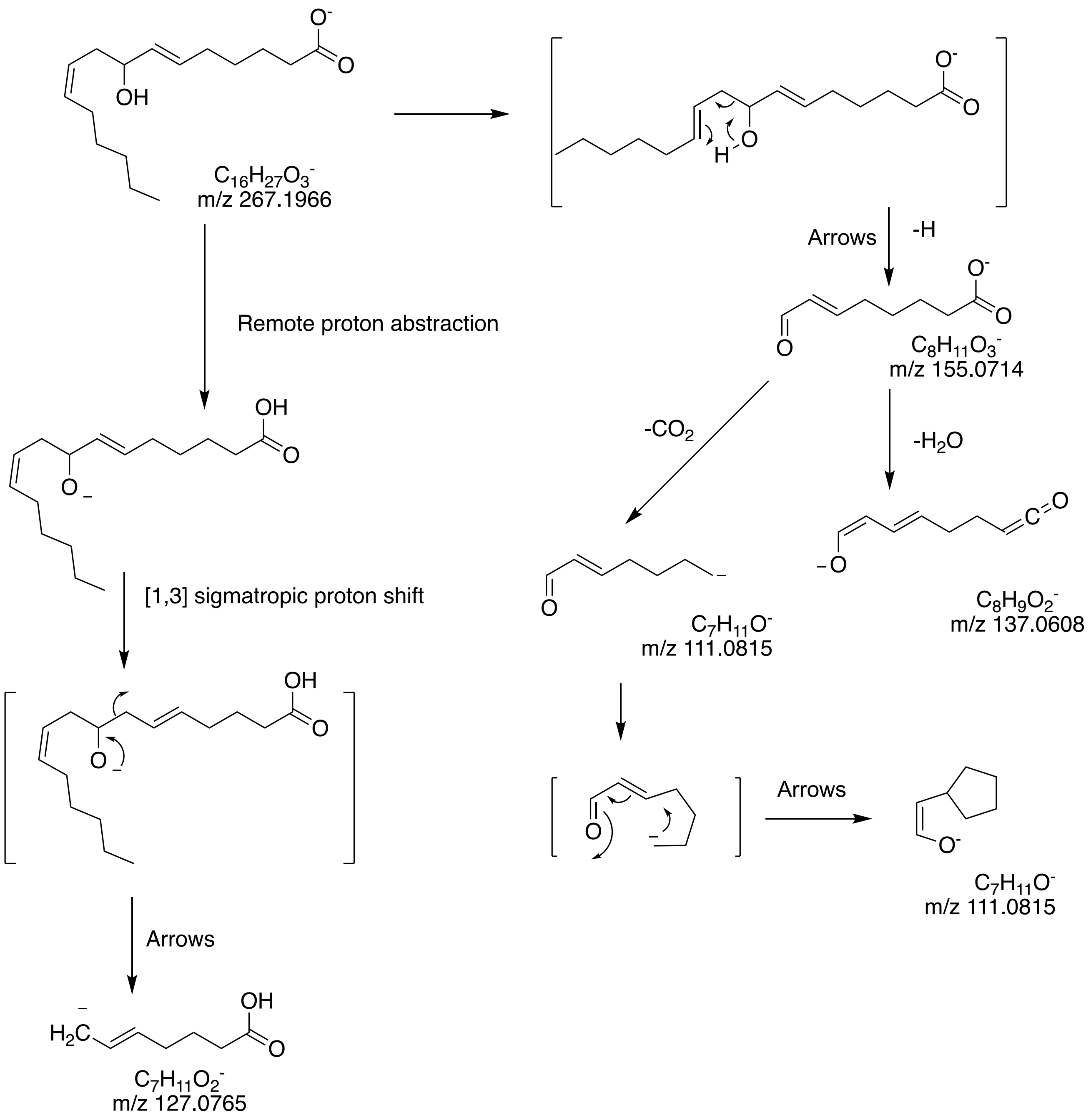

Supplementary Scheme 2. Proposed MS/MS for 8S-hydroxy-6E,10Z-hexadecadienoic acid (tetranor diene). The term "Arrows" is used to denote each of the steps where little one-sided arrows suggest electrons might move

Supplementary Figure 14

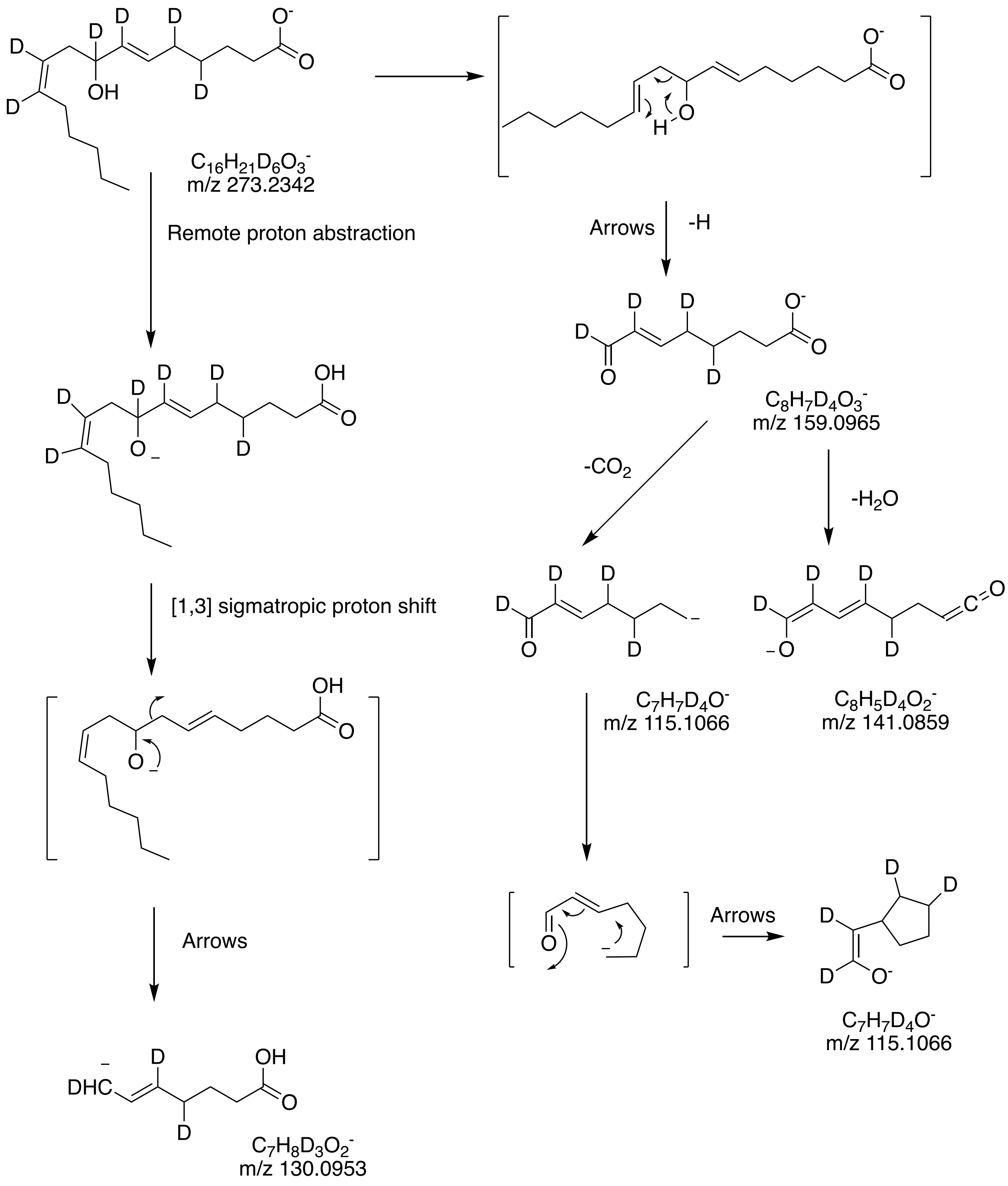

Supplementary Scheme 3. Proposed MS/MS for D_6-8S-hydroxy-6E,10Z-hexadecadienoic acid (tetranor diene).

Supplementary Figure 15. Proposed biochemical mechanism of β-oxidation for 12-HETE.

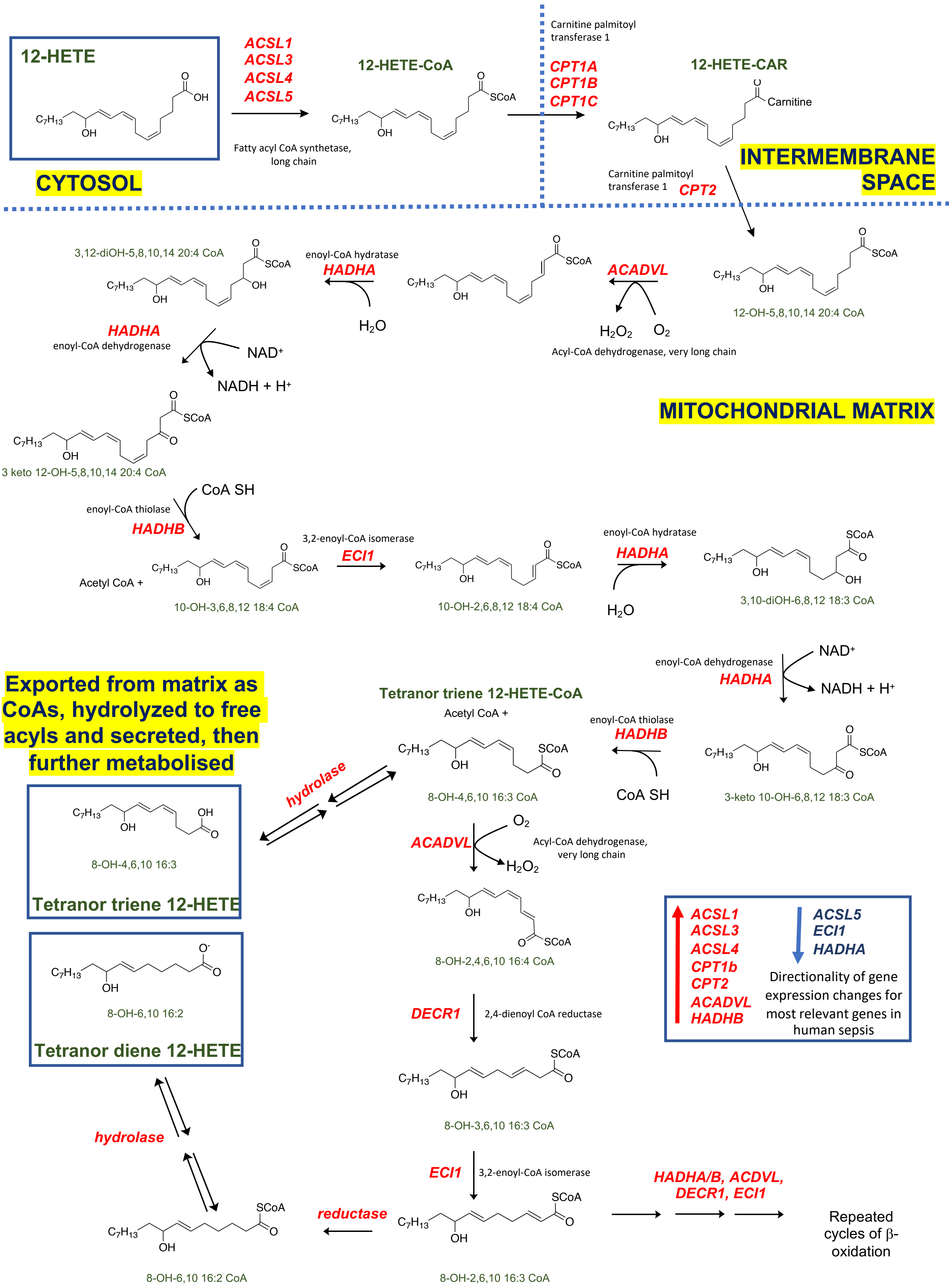

Supplementary Table 1

Lipids added to cells to simulate physiological amounts

These amounts are expressed in terms of ng/mouse which is estimated to have 100 ml peritoneal volume
Thus for calculating what to add to cells, they were added in these concentrations for the final experiment with whole blood cells

	ng/mouse	
median	low lipid	high lipid
5-HETE	0.20796525	0.4159305
8-HETE	0.223796	0.447592
9-HETE	0.08038325	0.1607665
11-HETE	0.27483475	0.5496695
12-HETE	7.3727075	14.745415
15-HETE	1.03487875	2.0697575
20-HETE	0.3686335	0.737267
11-HEPE	0.02583275	0.0516655
12-HEPE	0.27923875	0.5584775
15-HEPE	0.52596975	1.0519395
4-HDOHE	0.02050975	0.0410195
8-HDOHE	0.050181	0.100362
10-HDOHE	0.1987765	0.397553
11-HDOHE	0.059643	0.119286
13-HDOHE	0.1355335	0.271067
14-HDOHE	10.580795	21.16159
16-HDOHE	0.037497	0.074994
17-HDOHE	3.375567	6.751134
20-HDOHE	0.02456675	0.0491335
9-HODE	4.63153975	9.2630795
13-HODE	31.3015975	62.603195
9-HOTrE	0.37260025	0.7452005
13-HOTrE	11.3827	22.7654
15-HETrE	0.138236	0.276472
9-OxoODE	0.676628	1.353256
13-OxoODE	1.7746715	3.549343
12-OxoETE	1.64698925	3.2939785
15-OxoETE	0.2720885	0.544177
9,10-DiHOME	2.03603825	4.0720765
12,13-DiHOME	5.28231	10.56462
5,6-DiHETrE	0.05350925	0.1070185
8,9-DiHETrE	0.169896	0.339792
11,12-DiHETrE	0.2299905	0.459981
14,15-DiHETrE	0.3937595	0.787519
8,15-DiHETE	0.027859	0.055718
14,15-DiHETE	0.6634135	1.326827
17,18-DiHETE	1.1895955	2.379191
6-trans LTB4	0.0572235	0.114447
Mar-01	0.03122825	0.0624565
9(10)-EpOME	0.52882825	1.0576565
12(13)-EpOME	1.18597275	2.3719455
5(6)-EET	0.17895925	0.3579185
10(11)-EpDPA	0.00502125	0.0100425
19(20)-EpDPA	0.5217775	1.043555
PGD2	0.053451	0.106902
PGE1	0.02898425	0.0579685
PGE2	0.29952575	0.5990515
13,14-dihydro-15-keto PGD2	0.0087255	0.017451
8-iso PGE2	0.039122	0.078244
PGF2α	0.02432725	0.0486545
6-keto PGF1α	1.00629425	2.0125885
TXB2	0.188358	0.376716

Supplementary Table 2. Putative genes relevant to micothondrial uptake and β-oxidation

Symbol	Synonym(s)	Entrez Gene Name
Acaa1b	acetyl-Coenzyme A acyltransferase 1B, MGC29978, Thiolase b	acetyl-Coenzyme A acyltransferase 1B
ACAA2	0610011L04Rik, 3-ketoacyl coa thiolase, acetyl-CoA acyltransferase 2, acetyl-Coenzyme A acyltransferase 2 (mitochondrial 3-oxoacyl-Coenzyme A thiolase), AI255831, AI265397, D18Ertd240e, DSAEC, T1, THIOLASE	acetyl-CoA acyltransferase 2
ACAD10	2410021P16RIK, acyl-CoA dehydrogenase family member 10, acyl-Coenzyme A dehydrogenase family, member 10, MGC5601	acyl-CoA dehydrogenase family member 10
ACAD11	5730439E10Rik, acyl-CoA dehydrogenase family member 11, acyl-CoA dehydrogenase family, member 11, acyl-Coenzyme A dehydrogenase family, member 11, AI987948, RGD1306270	acyl-CoA dehydrogenase family member 11
Acad12	9330129D05Rik, acyl-Coenzyme A dehydrogenase family, member 12	acyl-Coenzyme A dehydrogenase family, member 12
ACAD8	2310016C19Rik, acyl-CoA dehydrogenase family member 8, acyl-CoA dehydrogenase family, member 8, acyl-Coenzyme A dehydrogenase family, member 8, AI786953, ARC42, RGD1564209	acyl-CoA dehydrogenase family member 8
ACAD9	2600017P15RIK, 4732402K02, acyl-CoA dehydrogenase family member 9, acyl-CoA dehydrogenase family, member 9, acyl-Coenzyme A dehydrogenase family, member 9, C630012L17RIK, MC1DN20, NPD002, NYGGF2	acyl-CoA dehydrogenase family member 9
ACADL	AA960361, ACAD4, ACOADA, acyl-CoA dehydrogenase long chain, acyl-CoA dehydrogenase, long chain, acyl-Coenzyme A dehydrogenase, long-chain, AU018452, C79855, LCAD, Long chain acyl-coa dehydrogenase	acyl-CoA dehydrogenase long chain
ACADM	ACAD1, Acetyl coenzyme a dehydrogenase, medium chain, Acetyl-Coenzyme A Dehydrogenase, acyl-CoA dehydrogenase medium chain, Acyl-CoA dehydrogenase, medium chain, acyl-Coenzyme A dehydrogenase, medium chain, AU018656, MCAD, MCADH, MEDIUM-CHAIN FATTY ACYL-COA DEHYDROGENASE	acyl-CoA dehydrogenase medium chain
ACADS	ACAD3, acyl-CoA dehydrogenase short chain, ACYL-COENZYME A DEHYDROGENASE, acyl-Coenzyme A dehydrogenase, short chain, AI196007, Bcd-1, Hdlq8, SCAD	acyl-CoA dehydrogenase short chain
ACADSB	2-MEBCAD, SBCAD, ACAD7, Acyl-CoA Dehydrogenase Short/Branched Chain, Short/Branched Chain Specific Acyl-CoA Dehydrogenase Mitochondrial, Acyl-Coenzyme A Dehydrogenase, Short/Branched Chain, 2-Methyl Branched Chain Acyl-CoA, 2-Methylbutyryl-Coenzyme A Dehydrogenase Dehydrogenase	Acyl-CoA dehydrogenase short/branched chain
ACADVL	ACAD6, acyl-CoA dehydrogenase very long chain, acyl-CoA dehydrogenase, very long chain, acyl-Coenzyme A dehydrogenase, very long chain, LCACD, VERY long-CHAIN FATTY ACYL-COA DEHYDROGENASE, VLACD, VLCAD	acyl-CoA dehydrogenase very long chain
ACSBG1	acyl-CoA synthetase bubblegum family member 1, BG, BG1, BGM, E230019G03Rik, Gm2069, GR-LACS, LPD, R75185	acyl-CoA synthetase bubblegum family member 1
ACSBG2	Acyl-CoA Synthetase Bubblegum Family Member, Long-Chain-Fatty-Acid--CoA Ligase ACSBG2, Arachidonate--CoA Ligase ACSBG2, Bubblegum-Related Protein, PRTD-NY3, BGR, PRTDNY3, BRGL	
ACSL1	Acas1, ACAS, ACS1, ACS, ACYL-COA SYNTHETASE, Acyl-coa synthetase 5, acyl-CoA synthetase long-chain family member 1, COAA, FACL1, FACL2, Facs1, FACS, Fatty acid coa ligase 2, Fatty Acid Coenzyme A Ligase, Long Chain 2, LACS2, LACS, LACS 1, Long Chain Acyl-CoA Synthetase 2, Long chain fatty acid CoA synthase	acyl-CoA synthetase long chain family member 1
ACSL3	2610510B12Rik, ACS3, ACYL-COA SYNTHETASE 3, acyl-CoA synthetase long-chain family member 3, C85929, FACL3, LACS3, PRO2194	acyl-CoA synthetase long chain family member 3
ACSL4	9430020A05Rik, ACS4, acyl-CoA synthetase long-chain family member 4, arachidonoyl-CoA synthetase, AU018108, FACL4, LACS4, Long-Chain Acyl-CoA Synthetase 4, MRX63, MRX68	acyl-CoA synthetase long chain family member 4
ACSL5	1700030F05Rik, ACS2, ACS5, Acyl CoA Synthetase 5, acyl-CoA synthetase long-chain family member 5, FACL5, Fatty Acid Coenzyme A Ligase, Long Chain5	acyl-CoA synthetase long chain family member 5
ACSL6	A330035H04Rik, ACS2, acyl-CoA synthetase long-chain family member 6, AW050338, DKFZp547D104, FACL6, LACS2, LACS5, LACS, LACS 6, Lacsl, long CHAIN ACYL COA SYNTHETASE6, mKIAA0837	acyl-CoA synthetase long chain family member 6
CPT1A	C730027G07, Carnithine palmitoyltransferase I, carnitine palmitoyltransferase 1A, carnitine palmitoyltransferase 1a, liver, Carnitine palmitoyltransferase i, CPT-Ia, CPT1, CPT1-L, CPTI, L-CPT1, Mitochondrial Carnitine O-palmitoyltransferase1	carnitine palmitoyltransferase 1A
CPT1B	carnitine palmitoyl transferase I, carnitine palmitoyltransferase 1B, carnitine palmitoyltransferase 1b, muscle, CPT1-M, CPTI, CPTI-M, M-CPT1, M-CPTI, MCCPT1	carnitine palmitoyltransferase 1B
CPT1C	Carnitine Palmitoyltransferase 1C, Carnitine O-Palmitoyltransferase 1, Carnitine O-Palmitoyltransferase I,j CPT1-B, CPTI-B, CATL1, CPT-IC, CPT1P, CPTIC, SPG73	
CPT2	AI323697, Carnitine o-palmitoyltransferase, Carnitine o-palmitoyltransferase ii, carnitine palmitoyltransferase 2, Carnitine Palmitoyltransferase II, CPT1, CPTASE, CPTII, IIAE4, Mitochondrial carnitine palmitoyltransferase II	carnitine palmitoyltransferase 2
DECR1	1200012F07Rik, 2,4 Dienoyl Reductase, 2,4-Dienoyl coa reductase, 2,4-dienoyl CoA reductase 1, mitochondrial, 2,4-dienoyl-CoA reductase 1, DECR, NADPH, SDR18C1	2,4-dienoyl-CoA reductase 1
ECHS1	C80529, ECHS1D, enoyl Coenzyme A hydratase, short chain, 1, mitochondrial, Enoyl-CoA hydratase, enoyl-CoA hydratase, mitochondrial-like, enoyl-CoA hydratase, short chain 1, Enoyl-CoA hydratase, short-chain, 1, mitochondrial, LOC100911186, SCEH	enoyl-CoA hydratase, short chain 1
ECI1	Enoyl-CoA Delta Isomerase 1, Dodecenoyl-Coenzyme A Delta Isomerase (3,2 Trans-Enoyl-Coenzyme A Isomerase), Enoyl-CoA Delta Isomerase 1, Mitochondrial, Delta(3),Delta(2)-Enoyl-CoA Isomerase, D3,D2-Enoyl-CoA, DCI Dodecenoyl-CoA Isomerase 2 4 Isomerase,	
ECI2	ACBD2, dJ1013A10.3, DRS-1, enoyl-CoA delta isomerase 2, enoyl-CoA ╬┤ isomerase 2, enoyl-Coenzyme A delta isomerase 2, enoyl-Coenzyme A ╬┤ isomerase 2, HCA88, PECI, Peroxisomal delta3, delta2-enoyl-Coenzyme A isomerase, Peroxisomal enoyl-coa isomerase	enoyl-CoA delta isomerase 2
HADH	3 Hydroxyacyl Coenzyme A Dehydrogenase, 3-hydroxyacyl-coa dehydrogenase, AA409008, AU019341, AW742602, Beta-3 Hydroxyacyl Coenzyme A Dehydrogenase, HAD, HADH1, HADHSC, HCDH, HHF4, hydroxyacyl-CoA dehydrogenase, hydroxyacyl-Coenzyme A dehydrogenase, MSCHAD, SCHAD, 3 Hydroxyacyl Coenzyme A Dehydrogenase	hydroxyacyl-CoA dehydrogenase
HADHA	C77020, ECHA, Enoyl-CoA Hydratase, GBP, HADH, hydroxyacyl-CoA dehydrogenase trifunctional multienzyme complex subunit alpha, hydroxyacyl-CoA dehydrogenase trifunctional multienzyme complex subunit, hydroxyacyl-Coenzyme A dehydrogenase/3-ketoacyl-Coenzyme A thiolase/enoyl-Coenzyme A hydratase (trifunctional protein), alpha subunit, hydroxyacyl-Coenzyme A dehydrogenase/3-ketoacyl-Coenzyme A thiolase/enoyl-Coenzyme A hydratase (trifunctional protein), LCEH, LCHAD, Mitochondrial long chain enoyl CoA hydratase, Mitochondrial trifunctional protein alpha, MTPA, RGD1560655, TP-ALPHA	hydroxyacyl-CoA dehydrogenase trifunctional multienzyme complex subunit alpha
HADHB	3-ketoacyl-CoA thiolase, 3-Ketoacyl-CoA Thiolase B, 4930479F15Rik, A hydratase (trifunctional protein), beta subunit, A hydratase (trifunctional protein), ECHB, hydroxyacyl-CoA dehydrogenase trifunctional multienzyme complex subunit beta, hydroxyacyl-CoA dehydrogenase trifunctional multienzyme complex subunit, hydroxyacyl-Coenzyme A dehydrogenase/3-ketoacyl-Coenzyme A thiolase/enoyl-Coenzyme A hydratase (trifunctional protein), beta subunit, hydroxyacyl-Coenzyme A dehydrogenase/3-ketoacyl-Coenzyme A thiolase/enoyl-Coenzyme A hydratase (trifunctional protein), Kat, Mitochondrial long chain 3-ketoacyl-CoA thiolase, MSTP029, MTPB, TP-BETA,	hydroxyacyl-CoA dehydrogenase trifunctional multienzyme complex subunit beta
SLC25A20	CACT, CAC	solute carrier family 25 (carnitine/acylcarnitine translocase), member 20
SLC27A1	ACSVL5, FATP, FATP-1, solute carrier family 27 (fatty acid transporter), member 1, solute carrier family 27 member 1	solute carrier family 27 member 1
SLC27A4	ACSVL4, BB144259, FATP4, IPS, solute carrier family 27 (fatty acid transporter), member 4, solute carrier family 27 member 4	solute carrier family 27 member 4
SLC27A6	4732438L20, 4732438L20Rik, ACSVL2, FACVL2, FATP6, solute carrier family 27 (fatty acid transporter), member 6, solute carrier family 27 member 6, VLCS-H1	solute carrier family 27 member 6

Supplementary Table 3. Primers used for macrophage polarization analysis

Gene	Forward Primer	Reverse Primer
TNF	AATGGCCTCCCTCTCATCAG	GTGGTTTGCTACGACGTGG
CD11c	ACGCTTACCTGGGTTACTCC	AAGATGACAACCTTCCCCGT
Arg1	ACAAGACAGGGCTCCTTTC A	TGCCGTGTTCACAGTACTCT
B-actin	TGGCACCACACCTTCTACAA	AGGTCTCAAACATGATCTGGGT

Supplementary Table 4. MRM and instrument parameters for HETE metabolites

Analyte	Internal Standard (IS)	Declustering potential (DP)	Collision energy (CE)	Molecular mass (g/ mol)	Transition	
					Q1 (m/z)	Q3 (m/z)
Tetranor 12-HETE triene	15-HETE d8	-52	-30	266.5	265.2	109.1
Tetranor 12-HETE diene	15-HETE d8	-52	-30	268.5 (putative)	267.1	155.1
Tetranor 12(S)-HETE d8 triene	15-HETE d8	-52	-30	273.5 (putative)	272.2	169.1
Tetranor 12(S)-HETE d8 diene_1	15-HETE d8	-52	-30	275.5 (putative)	274.2	131.1
12-HETE	15-HETE d8	-52	-30	320.5	319.2	184.2
12-HETE d8	15-HETE d8	-52	-30	328.5	327.2	184.2

Supplementary Table 5. mtDNA primers for copy number assessment

Assay	Species	Target	Direction	Sequence	product
mtDNA copy number	Mouse	18s Ribosome	Forward	AAACGGCTACCACATCCAAG	112 bp
	Mouse	18s Ribosome	Reverse	CAATTACAGGGCCTCGAAAG	
	Mouse	mouse mtDNA	Forward	CCCCAGCCATAACACAGTATCAAAC	201 bp
	Mouse	mouse mtDNA	Reverse	GCCCAAAGAATCAGAACAGATGC	
mtDNA Damage	Mouse	mouse mtDNA	Forward short primer (short PCR product)	GCAAATCCATATTCATCCTTCTCAAC	80 bp
	Mouse	mouse mtDNA	Forward long primer (long PCR product)	CCCAGCTACTACCATCATTCAAGTAG	16.2 kb

Supplementary Table 6. MRM and instrument parameters for oxidized phospholipids

MRM	Assignment	Retention Time (mins)
738.6/179.1	PE P-16:0_12-HETE	12.40
754.6/179.1	PE 16:0_12-HETE	11.80
764.6/179.1	PE P-18:1_12-HETE	12.63
766.6/179.1	PE P-18:0_12-HETE	13.96
780.6/179.1	PE 18:1_12-HETE	12.05
780.6/179.1	PC 16:1_12-HETE	10.51
782.6/179.1	PE 18:0_12-HETE	13.31
782.6/179.1	PE 16:0_12-HETE	11.72
808.7/179.1	PE 18:1_12-HETE	11.95
810.7/179.1	PE 18:0_12-HETE	13.27

Supplementary Table 7. MRM transitions and MS parameters for FFA and the deuterated internal standards utilised (same MS settings)

Compound (carbons: double bonds)	DP (volts)	CE (volts)	Q1 m/z	Q3 m/z	Internal std (IST)	Q1 m/z (IST)	Q3 m/z (IST)
Palmitic acid (16:0)	-137	-44	390.4	137	Palmitic acid-d2	392.44	137
Stearic acid (18:0)	-140	-50	418.48	137	Stearic acid-d35	453.69	137
Arachidonic acid (20:4)	-145	-42	438.47	137	Arachidonic acid-d8	446.52	137
