# Supplementary material for: Oxylipin metabolism is controlled by mitochondrial β-oxidation during bacterial inflammation
Source: Nat Commun. 2022 Jan 10;13:139. doi: 10.1038/s41467-021-27766-8 (PMC8748967; doi:10.1038/s41467-021-27766-8)
Supplement: Supplementary file 3 — Description of additional Supplementary File [file 41467_2021_27766_MOESM3_ESM.pdf]

Description of additional supplementary data files

Supplementary Dataset 1: Oxylipin abbreviations and enzymatic source

Supplementary Dataset 2: Full parameters for LC/MS/MS oxylipin assay
