# Supplementary material for: Oxylipin metabolism is controlled by mitochondrial β-oxidation during bacterial inflammation
Source: Nat Commun. 2022 Jan 10;13:139. doi: 10.1038/s41467-021-27766-8 (PMC8748967; doi:10.1038/s41467-021-27766-8)
Supplement: Supplementary file 6 — Reporting Summary [file 41467_2021_27766_MOESM6_ESM.pdf]

nature research

Corresponding author(s): Valerie O'Donnell

Last updated by author(s): 12th Oct 2021

Reporting Summary

Nature Research wishes to improve the reproducibility of the work that we publish. This form provides structure for consistency and transparency in reporting. For further information on Nature Research policies, see our Editorial Policies and the Editorial Policy Checklist.

Statistics

For all statistical analyses, confirm that the following items are present in the figure legend, table legend, main text, or Methods section.

n/a	Confirmed	
☐	☒	The exact sample size (*n*) for each experimental group/condition, given as a discrete number and unit of measurement
☐	☒	A statement on whether measurements were taken from distinct samples or whether the same sample was measured repeatedly
☐	☒	The statistical test(s) used AND whether they are one- or two-sided<br>*Only common tests should be described solely by name; describe more complex techniques in the Methods section.*
☐	☒	A description of all covariates tested
☐	☒	A description of any assumptions or corrections, such as tests of normality and adjustment for multiple comparisons
☐	☒	A full description of the statistical parameters including central tendency (e.g. means) or other basic estimates (e.g. regression coefficient) AND variation (e.g. standard deviation) or associated estimates of uncertainty (e.g. confidence intervals)
☐	☒	For null hypothesis testing, the test statistic (e.g. *F*, *t*, *r*) with confidence intervals, effect sizes, degrees of freedom and *P* value noted<br>*Give P values as exact values whenever suitable.*
☒	☐	For Bayesian analysis, information on the choice of priors and Markov chain Monte Carlo settings
☐	☒	For hierarchical and complex designs, identification of the appropriate level for tests and full reporting of outcomes
☒	☐	Estimates of effect sizes (e.g. Cohen's *d*, Pearson's *r*), indicating how they were calculated

Our web collection on statistics for biologists contains articles on many of the points above.

Software and code

Policy information about availability of computer code

Data collection

Seahorse Analyser used the manufacturer's installed software, Wave 2.3/4.
Analyst Software (V1.6) was used for collection of lipidomics data

Data analysis

MultiQuant software (version 3.0.2, AB Sciex Framingham, MA, U.S.A.) for integration of lipidomics data.
Heatmaps were generated using Pheatmap in R (V1)
Statistical analysis was performed using Prizm (7-9) or Excel (staff members used Excel 2013, 2016 and office 365 versions)

Attune NxT software V2 was used for Flow cytometry

All datasets were processed in R and using packages from Bioconductor. Affymetrix microarray data (GSE69607, GSE5099) were normalized and processed using Limma and Oligo Bioconductor packages
Paired-end reads from Illumina sequencing (GSE53053, GSE84517) were trimmed with Trim Galore and assessed for quality using FastQC, using default parameters..
Versions of informatics software are listed below.

R	3.6.3
limma	3.40.6
oligo	1.48.0
lumi	2.36.0
DESeq2	1.24.0
TrimGalore	0.5.0
FastQC	0.11.8
featureCounts	2.0.0

STAR 2.7.0
Bowie2 2.3.4.3

Reads were mapped to the reference sequence (human GRCh38 or mouse GRCm38) reference genome using STAR and counts were assigned to transcripts using featureCounts with the Ensembl gene build GTF (GRCh38.96 or GRCm38.84)
Differential gene expression analyses used the DESeq2 package

One way ANOVA used https://astatsa.com/OneWay_Anova_with_TukeyHSD/ (no version available).

For manuscripts utilizing custom algorithms or software that are central to the research but not yet described in published literature, software must be made available to editors and reviewers. We strongly encourage code deposition in a community repository (e.g. GitHub). See the Nature Research guidelines for submitting code & software for further information.

Data

Policy information about availability of data

All manuscripts must include a data availability statement. This statement should provide the following information, where applicable:

- Accession codes, unique identifiers, or web links for publicly available datasets
- A list of figures that have associated raw data
- A description of any restrictions on data availability

All data used for the study are available in the Supplementary File. Several published transcriptomic datasets were used in Figures 5-7. These are all publicly available at GEO (GSE53053, GSE69607, GSE84517, GSE46903, GSE35449, GSE5099), EMBL-EBIE-MTAB-10087, GSE25504. There are no restrictions on use of that data, which doesn't belong to us but is publicly available.

Field-specific reporting

Please select the one below that is the best fit for your research. If you are not sure, read the appropriate sections before making your selection.

☒ Life sciences ☐ Behavioural & social sciences ☐ Ecological, evolutionary & environmental sciences

For a reference copy of the document with all sections, see nature.com/documents/nr-reporting-summary-flat.pdf

Life sciences study design

All studies must disclose on these points even when the disclosure is negative.

Sample size	Sample sizes were chosen based on previous experience conducting cellular and animal model studies in our laboratory. These include studies conducted over many years, for example in PMID18941242. Power calculations have been conducted previously for other murine experiments, but were not specifically undertaken here since we did not know the variability of the specific experiments in advance and pilot studies weren't undertaken. Where data was used from published sources (e.g. GEO datasets), the sample size would have been determined by other investigators as part of their work. We analysed all available datasets we could find and did not restrict to a specific number for any reasons.
Data exclusions	No data were excluded from analysis unless known human errors occurred during sample processing which rendered these samples unusable. Outliers are always included in all studies.
Replication	For all experiments the number of biological and technical replicates used for the experiments are provided in figure legends<br>Human cell experiments were conducted on 3 individual donors. All three donors are shown in the figures.
Randomization	Animals were randomly assigned to groups to receive drugs, there were no specific assignments to any experimental groups Cultured cells were randomly assigned to wells of plates.
Blinding	Investigators weren't blinded when carrying out experiments, since they needed to have effective oversight of sample handling when making additions of drugs or inflammatory activators. Our studies did not involve behavioral assessment or other types of modality for which unblinded studies can induce a high biases. Our analyses all generate objective outcomes that are not subject to observer bias.<br>Some data was obtained from other groups, e.g. transcriptional data from GEO, and we have no information on blinding for these studies.

Reporting for specific materials, systems and methods

We require information from authors about some types of materials, experimental systems and methods used in many studies. Here, indicate whether each material, system or method listed is relevant to your study. If you are not sure if a list item applies to your research, read the appropriate section before selecting a response.

Materials & experimental systems

n/a	Involved in the study
	[x] Antibodies
	[x] Eukaryotic cell lines
[x]	Palaeontology and archaeology
	[x] Animals and other organisms
	[x] Human research participants
[x]	Clinical data
[x]	Dual use research of concern

Methods

n/a	Involved in the study
[x]	ChIP-seq
	[x] Flow cytometry
[x]	MRI-based neuroimaging

Antibodies

Antibodies used

F4/80 (BD Horizon, BV421 Rat Anti-Mouse F4/80, Clone T45-2342, Cat.: 565411)
F4/80 (BD Horizon, BV421 Rat Anti-Mouse F4/80, Clone T45-2342, Cat.: 565411
CD11b-allophycocyanin antibodies (BD Pharmingen, Clone M1/70, PE Rat Anti-Mouse, Cat 557397)
CD16/CD32 Rat anti-Mouse, unlabeled, Clone: 2.4G2, BD 553142
anti-CD15 BV510 (1:50 in 0.5 ml PBS, W6D3, Becton Dickinson, 563141)
anti-CD8 BV-711 (RPA-T8), BioLegend, cat # 301044, used at 1:100 dilution in cells.
anti-CD4 BV605 (OKT4), BioLegend, cat # 317438, used at 1:25 dilution in cells
anti-CD3 Percp (SK7). BioLegend, cat # 344814, used at 1:50 dilution in cells.
anti-TNF APC-Vio770 (cA2) – Miltenyi Biotec, cat # 130-120-629, used at 1:50 dilution in cells.
Anti-mouse IgG-κ compensation particles were used for single stain compensation controls – BD Biosciences, cat # 552843, 1 drop of beads per 100ul staining buffer. 50ul used per compensation control tube.

Validation

F4/80 (BD Horizon, BV421 Rat Anti-Mouse F4/80, Clone T45-2342, Cat.: 565411): validation was caarried out as described here (https://www.bdbiosciences.com/en-gb/products/reagents/flow-cytometry-reagents/research-reagents/single-color-antibodies-ruo/bv421-rat-anti-mouse-f4-80.565411) and used spleen and peritoneal cells.

CD11b-allophycocyanin antibodies (BD Pharmingen, Clone M1/70, PE Rat Anti-Mouse, Cat 557397): validation used bone marrow derived myeloid cells as described here: https://www.bdbiosciences.com/en-us/products/reagents/flow-cytometry-reagents/research-reagents/single-color-antibodies-ruo/apc-rat-anti-cd11b.553312

CD16/CD32 Rat anti-Mouse, unlabeled, Clone: 2.4G2, BD 553142: validation used mouse spleen as outlined here: https://www.bdbiosciences.com/en-us/products/reagents/flow-cytometry-reagents/research-reagents/single-color-antibodies-ruo/purified-rat-anti-mouse-cd16-cd32-mouse-bd-fc-block.553142

anti-CD15 BV510 (1:50 in 0.5 ml PBS, W6D3, Becton Dickinson, 563141): Validation used human peripheral blood granulocytes as outlined here: https://www.bdbiosciences.com/en-us/products/reagents/flow-cytometry-reagents/research-reagents/single-color-antibodies-ruo/bv510-mouse-anti-human-cd15.563141

anti-CD8 BV-711 (RPA-T8)
Validation used human peripheral blood monocytes and is shown here: https://www.biolegend.com/en-gb/products/brilliant-violet-711-anti-human-cd8a-antibody-7929

anti-CD4 BV605 (OKT4). Validation used human peripheral lymphocytes, and is shown here: https://www.biolegend.com/en-gb/products/brilliant-violet-605-anti-human-cd4-antibody-7820

anti-CD3 Percp (SK7).
Validation used human peripheral lymphocytes, and is shown here: https://www.biolegend.com/en-gb/products/percp-anti-human-cd3-antibody-6933

anti-TNFa APC-Vio770 (cA2)
Validation used human peripheral mononuclear cells, and is described here: https://www.miltenyibiotec.com/GB-en/products/tnf-a-antibody-anti-human-ca2.html#gref

Anti-mouse IgG-κ compensation particles
Validation is not described on their websites. This is a control reagent for setting compensation levels only.

Eukaryotic cell lines

Policy information about cell lines

Cell line source(s)	RAW264.7 and HEK293.2 cells which we have cultured in our laboratory for many years. RAW cells either overexpressing Alox15 or with Cpt1a knockdown were generated.
Authentication	No authentication process was used was used for the wild type cells, but the RAW cells were validated for overexpression of 12/15-LOX by analysis of gene expression and lipid products. Cpt1a knockdown was confirmed by PCR. These data are in the manuscript.
Mycoplasma contamination	Cell lines have been tested for mycoplasma routinely for many years, and always been negative. Mycoplasma free stocks were resuscitated and cultured for use.
Commonly misidentified lines (See ICLAC register)	None

Animals and other organisms

Policy information about studies involving animals; ARRIVE guidelines recommended for reporting animal research

Laboratory animals	C57BL/6 mice were used, all female between 7-13 weeks, Mouse breeders were housed in isolators (barrier) and experimental mice in individually ventilated cages with 12-h light/dark cycles and controlled temperature (20 – 22 °C) (specific pathogen free, SPF). Access to water and standard chow was ad libitum, and humidity was atmospheric, and not specifically regulated.
Wild animals	No wild animals were used in the study
Field-collected samples	No field animals were used in the study
Ethics oversight	All mouse experiments were performed in accordance with the United Kingdom Home Office Animals (Scientific Procedures) Act of 1986 (P05K6A456) and for peritoneal models under (UK Home Office PPL 30/2938, and PPL P05D6A456).

Note that full information on the approval of the study protocol must also be provided in the manuscript.

Human research participants

Policy information about studies involving human research participants

Population characteristics	Data from human studies that was included all came from previously published research that is fully referenced in our paper. Workplace volunteers donated blood and were male or female, healthy, between the ages of 25-55, with no genotype information.
Recruitment	Recruitment was by local advertisement. Given the comparisons are within the samples from individuals, e.g. each person acted as their own control, not between individuals, there should be no impact of selection bias
Ethics oversight	Human blood from healthy workplace volunteers was obtained with ethical approval from School of Medicine (SMREC 16/02) with informed consent. There was no participant compensation.

Note that full information on the approval of the study protocol must also be provided in the manuscript.

Flow Cytometry

Plots

Confirm that:

- [x] The axis labels state the marker and fluorochrome used (e.g. CD4-FITC).
- [x] The axis scales are clearly visible. Include numbers along axes only for bottom left plot of group (a 'group' is an analysis of identical markers).
- [x] All plots are contour plots with outliers or pseudocolor plots.
- [x] A numerical value for number of cells or percentage (with statistics) is provided.

Methodology

Sample preparation	Cells were labelled and analysed live for flow cytometric sorting of Cpt1a knockdown RAW264 cells via GFP expression and to identify APF fluorescence in human monocytes and neutrophils.
Instrument	The ThermoFisher Attune NXT was used for human blood analysis and the BD FACS Aria II was used for RAW264 cell sorting (100um nozzle)

Software	BD FACSDiva was used for RAW264 sorting, and the Attune NXT software was used for human blood sample collection. BD FlowJo was used for analysis.
Cell population abundance	No sorting was performed<br>GFP positive cells were approximately 50% of total live RAW264 cells, Neutrophils were approximately 45-60% of white blood cells and between 10 and 50% of them were APF positive, Monocytes made up approximately 5-10% of white blood cells and >80% of them were AFP positive.
Gating strategy	Positively infected RAW264 singlet cells (with knockdown and control lentiviruses) were discriminated by GFP expression. Singlet neutrophils were distinguished by their FSc/SSc profile and by from eosinophils via high expression of CD15. Singlet monocytic cells were distinguished by their intermediate SSc profile and low expression of CD15.

☒ Tick this box to confirm that a figure exemplifying the gating strategy is provided in the Supplementary Information.
